# IL-23 stabilizes an effector T_reg_ cell program in the tumor microenvironment

**DOI:** 10.1038/s41590-024-01755-7

**Published:** 2024-02-14

**Authors:** Tobias Wertheimer, Pascale Zwicky, Lukas Rindlisbacher, Colin Sparano, Marijne Vermeer, Bruno Marcel Silva de Melo, Claudia Haftmann, Tamina Rückert, Aakriti Sethi, Stefanie Schärli, Anna Huber, Florian Ingelfinger, Caroline Xu, Daehong Kim, Philipp Häne, André Fonseca da Silva, Andreas Muschaweckh, Nicolas Nunez, Sinduya Krishnarajah, Natalie Köhler, Robert Zeiser, Mohamed Oukka, Thomas Korn, Sonia Tugues, Burkhard Becher

**Affiliations:** 1 Department of Inflammation Research, Institute of Experimental Immunology, University of Zurich, Zurich, Switzerland; 2 Department of Pharmacology, Center for Research in Inflammatory Diseases, Ribeirao Preto Medical School, University of Sao Paulo, Sao Paulo, Brazil; 3 Department of Internal Medicine I, Hematology, Oncology, and Stem Cell Transplantation, Faculty of Medicine, Medical Centre, University of Freiburg, Freiburg, Germany; 4 Institute for Experimental Neuroimmunology, Klinikum Rechts der Isar, Technical University of Munich, Munich, Germany; 5 Centre for Integrative Biological Signalling Studies (CIBSS), University of Freiburg, Freiburg, Germany; 6 Department of Immunology, University of Washington, Seattle, WA USA; 7 Munich Cluster for Systems Neurology (SyNergy), Munich, Germany

**Keywords:** Tumour immunology, Interleukins, Immunosuppression

## Abstract

Interleukin-23 (IL-23) is a proinflammatory cytokine mainly produced by myeloid cells that promotes tumor growth in various preclinical cancer models and correlates with adverse outcomes. However, as to how IL-23 fuels tumor growth is unclear. Here, we found tumor-associated macrophages to be the main source of IL-23 in mouse and human tumor microenvironments. Among IL-23-sensing cells, we identified a subset of tumor-infiltrating regulatory T (T_reg_) cells that display a highly suppressive phenotype across mouse and human tumors. The use of three preclinical models of solid cancer in combination with genetic ablation of *Il23r* in T_reg_ cells revealed that they are responsible for the tumor-promoting effect of IL-23. Mechanistically, we found that IL-23 sensing represents a crucial signal driving the maintenance and stabilization of effector T_reg_ cells involving the transcription factor Foxp3. Our data support that targeting the IL-23/IL-23R axis in cancer may represent a means of eliciting antitumor immunity.

## Abstract (web summary)

IL-23 promotes tumor growth in preclinical cancer models and correlates with adverse clinical outcomes. Here, Becher and colleagues find that IL-23 produced by tumor-associated macrophages stabilizes T_reg_ cell identity, promoting immunosuppression and tumor growth.

## Main

Regulatory T (T_reg_) cells are a functionally distinct T cell population expressing the transcription factor Foxp3 that are critically involved in maintaining immune homeostasis^1^. Like conventional T cells, T_reg_ cells can undergo functional activation after T cell antigen receptor (TCR) stimulation, converting naive to highly suppressive effector T_reg_ (eT_reg_) cells^2^. This cellular subset is marked by augmented expression of Foxp3, CTLA-4, interleukin-10 (IL-10), ICOS and TIGIT (among others) and represents the dominant T_reg_ cell subpopulation in non-lymphoid tissues and tumors^3,4^.

In the context of cancer, both mouse and human tumor microenvironments (TMEs) are enriched with T_reg_ cells, contributing to an immunosuppressive niche suppressing antitumor immune responses and limiting therapeutic success of immunotherapy^5,6^. Although generalized T_reg_ cell depletion has proven to be efficacious in most preclinical tumor models^7–9^, it also induces systemic inflammation^10^. Consequently, strategies to reduce their suppressive capacities or destabilize T_reg_ cells specifically in the TME are attractive targets for cancer immunotherapy.

IL-23 is a member of the IL-12 superfamily of cytokines, which is primarily produced by cells of the mononuclear phagocyte system^11^. IL-23 drives the pathophysiology of immune disorders, such as psoriasis and inflammatory bowel disease, by inducing a pathogenic lymphocyte program promoting tissue inflammation^12,13^. Paradoxically, in the context of cancer, IL-23 exerts tumor-promoting functions. As such, ablation of both IL-23 or its receptor leads to reduced tumor burden^14–17^. The tumor-promoting effects of IL-23 appear to be independent of IL-17 (ref. ^16^), and ablation of IL-23 is associated with an enhanced infiltration of CD8^+^ T cells, natural killer (NK) cells and T_reg_ cells^14,15,17^.

To understand the mechanisms underpinning the protumorigenic functions of IL-23, we systematically interrogated both the cellular sources and sensors of IL-23 in mouse and human TMEs. We identified tumor-associated macrophages (TAMs) as the main cellular source of IL-23 and tumor-infiltrating T_reg_ cells as an IL-23 receptor (IL-23R)-expressing cell type. IL-23 stabilizes eT_reg_ cell identity and Foxp3 expression, thus enhancing immunosuppression, resulting in decreased antitumor immunity. Our findings render the IL-23/IL-23R axis a promising therapeutic target for the selective destabilization of tumor-infiltrating eT_reg_ cells for cancer immunotherapy.

## Results

### IL-23R marks a highly activated T_reg_ subset in the mouse TME

**Fig. 1:**
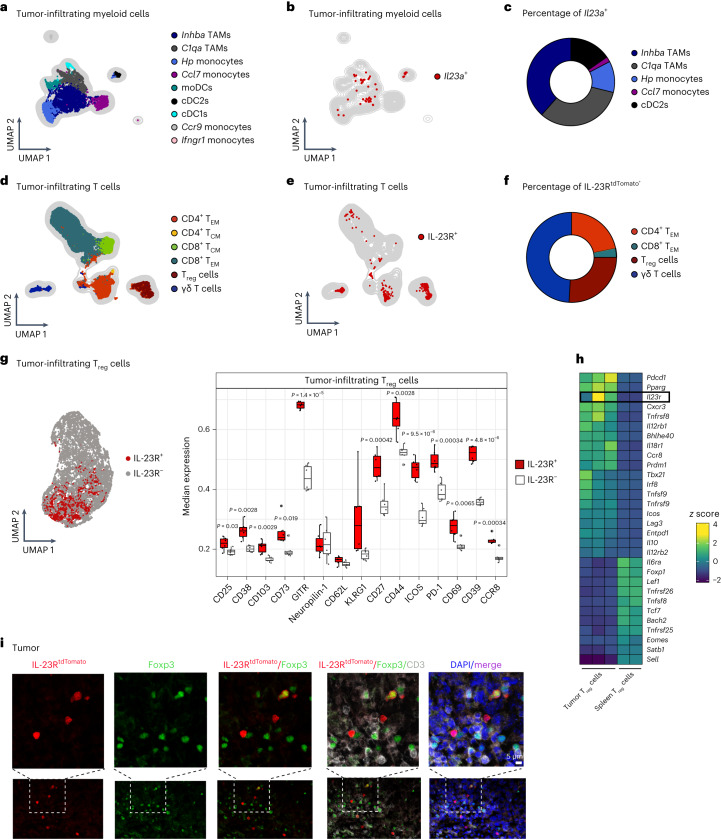
IL-23R marks a highly suppressive T_reg_ cell subset in the mouse TME. **a**–**c**, Analysis of a myeloid cell scRNA-seq dataset from mouse B16 tumors^18^ (GSE188548; WT tumor). **a**, UMAP depicting tumor-infiltrating myeloid cell clusters. **b**, UMAP displaying *Il23a*^+^ myeloid cells. **c**, Pie chart displaying the frequencies of myeloid cell subsets among total *Il23a*^+^ myeloid cells. **d**–**g**, *Foxp3*^DTR-GFP^IL-23R^tdTomato^ mice were inoculated intradermally (i.d.) with B16 tumors. TILs were analyzed by flow cytometry on day 14. Data are shown from one representative experiment out of two independent experiments with *n* = 5–6 biologically independent animals. **d**, UMAP with overlaid FlowSOM clustering (gated on CD45^+^TCRβ^+^TCRγδ^+^ cells). **e**, UMAP displaying IL-23R^tdTomato+^ T cells. **f**, Pie chart depicting the frequencies of T cell subsets among total IL-23R^tdTomato+^ T cells. **g**, UMAP with overlaid FlowSOM clustering displaying IL-23R^tdTomato+^Foxp3^+^ and IL-23R^tdTomato–^Foxp3^+^ T_reg_ cell clusters (left). Box plots showing median expression of surface markers on IL-23R^+^ and IL-23R^–^ T_reg_ cells are shown on the right. Box plots display the median and interquartile range (IQR; 25–75%), with whiskers representing the upper and lower quartiles ± IQR. Statistical significance was calculated using two-tailed *t*-tests. **h**, Analysis of a bulk next-generation sequencing dataset of T_reg_ cells sorted from B16 tumors or spleens (Magnuson et al.^27^). A heat map depicting selected genes among the top 50 DEGs is shown. Expression of *Il23r* is highlighted. **i**, Immunofluorescence stainings of tumors from i.d. inoculated B16 tumor-bearing IL-23R^tdTomato^ mice showing Foxp3 (green), IL-23R^Tdtomato^ (red), CD3 (white), DAPI (blue) and merged signals (purple). Scale bar: 5 μm. Images shown (*n* = 4) are representative of two independent experiments; moDCs, monocyte-derived DCs; T_CM_, central memory T cells. Source data

**Extended Data Fig. 1:**
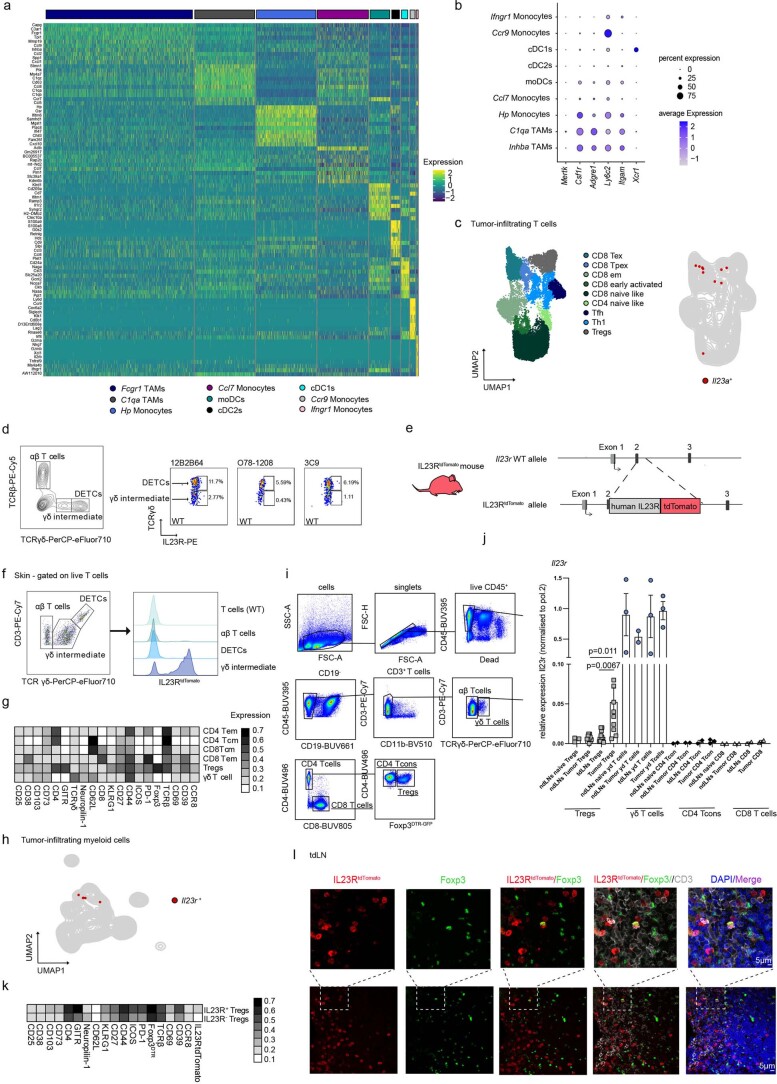
IL23R marks a highly suppressive Treg cell subset in the murine TME. **(a, b, h)** Myeloid cell-scRNAseq data from murine B16 tumors (Mujal et al. 2022, GSE188548). **(a)** Heatmap depicting the expression of cell-type-defining genes. **(b)** Dotplot showing the expression of selected linage markers. **(c)** scRNAseq data of murine pan-tumor T cells (Andreatta et al. 2022, E-MTAB-9274). UMAP displaying T cell subsets (left) or Il23a^+^ T cells (right). **(d)** Representative FACS plot (left) of steady state murine skin CD45^+^cells (left) (pregated on live, CD45^+^cells). Representative FACS plots (right, pregated on live, CD45^+^, TCRγδ^+^) with gates of positive signal of respective IL23R-PE antibody clones of dendritic epidermal T cells (DETCs) (upper gate) and γδ^intermediate^ T cells (lower gate). Displayed data are from one experiment with n = 4. **(e)** Schematic illustration of the IL23R^tdTomato^allele. **(f)** Representative FACS plot (left) of murine skin T cells and histograms (right) depicting IL23R^tdTomato^ expression among T cell subsets from IL23R^tdTomato^ mice and total T cells (‘T cells’) from WT mice. Data shown from one representative experiment out of two independent experiments with n = 3. **(g, k)** T cells from i.d. inoculated B16 tumors in Foxp3^DTR-GFP^ IL23R^tdTomato^ mice were analyzed by flow cytometry on day 14. Data shown from one representative experiment out of two independent experiments with n = 5-6. **(g)** Heatmap displaying marker expression among tumor-infiltrating T cell subsets. **(h)** UMAP displaying Il23r^+^ myeloid cells. **(i)** Gating strategy used to FACS-sort γδ, CD8^+^, CD4^+^ T cells and Foxp3^+^ Treg cells from Foxp3^DTR-GFP^ mice. **(j)** Bar graph depicting relative Il23r mRNA expression level (qPCR) in FACS sorted T cells from steady state LNs or tdLNs, ndLNs and tumors from i.d. inoculated B16 tumor-bearing Foxp3^DTR-GFP^ mice. Pooled data from three independent experiments. Biologically independent samples: n = 2: ndLNs: γδ T cells, naïve CD4 Tcon; ndLNs: naïve CD8; tdLNs: CD8, CD4 Tcon; Tumor: CD8. n = 3: ndLNs: naïve Treg cell, naïve γδ T cells, γδ T cells; Tumor: γδ T cells, CD4 Tcon. n = 8: ndLNs Tumor Treg cell. n = 10: tdLNs: Treg cell; Tumor: Treg cell. Data are displayed as mean +/- SEM. **(k)** Heatmap displaying relative marker expression among IL23R^+^ and IL23R^−^Treg cellclusters. **(l)** Immunofluorescence-stainings of tdLNs from i.d. inoculated B16-tumor bearing IL23R^tdTomato^ mice. Foxp3 (green), IL23R^tdTomato^ (red), CD3 (white) DAPI (blue), merged (purple). Images (n = 4) are representatives from 2 independent experiments. Source data

To identify the cellular sources of IL-23 in the TME, we investigated the expression of *Il23a* (encoding IL-23p19) in single-cell RNA-sequencing (scRNA-seq) data of tumor-infiltrating myeloid cells in the mouse B16-F10 tumor model^18^ (hereafter B16; Fig. 1a–c) and mouse pan-tumor T cells from 21 cancer entities^19^ (Extended Data Fig. 1c). We identified two TAM, four monocyte and three dendritic cell (DC) clusters (Fig. 1a and Extended Data Fig. 1a,b). Although the expression of *Il23a* was generally low, we assigned two TAM populations (Spp1^+^ and C1q^+^ TAMs) as the major *Il23a*-producing cells in the TME (Fig. 1b,c). In addition, monocytes (*Hp*^+^ and *Ccl7*^+^ monocytes) and conventional type 2 DCs (cDC2s) contributed to the total *Il23a* expression in the TME (Fig. 1b,c), with only negligible amounts in tumor-infiltrating T cells (TILs; Extended Data Fig. 1c).

In line with previous findings^20^, we found that flow cytometry using anti-IL-23R, previously used by others^21–23^, failed to faithfully detect IL-23R as no IL-23R expression was observed in skin γδ^intermediate^ (Vγ4/Vγ6) T cells (Extended Data Fig. 1d)^24^. Therefore, we generated an IL-23R reporter mouse strain where endogenous mouse *Il23r* was replaced by a gene construct composed of human *IL23R* cDNA and tdTomato (hereafter IL-23R^tdTomato^ mice; Extended Data Fig. 1e). In line with previous studies^24^, we detected IL-23R^tdTomato^ signal in γδ^intermediate^ T cells but not γδ^high^ (Vγ5 T cells/dendritic epidermal T cells) or αβ T cells of steady-state mouse skin (Extended Data Fig. 1f). To reliably capture all T cell subsets including T_reg_ cells within the TME, we crossed IL-23R^tdTomato^ mice with *Foxp3*^DTR-GFP^ mice and analyzed TILs (CD45^+^TCRβ^+^TCRγδ^+^ cells) after challenge with B16 melanoma (Fig. 1d–f and Extended Data Fig. 1g). Uniform manifold approximation and projection (UMAP) dimensionality reduction followed by FlowSOM metaclustering^25,26^ revealed that, besides γδ T cells and CD4^+^ effector memory T (T_EM_) cells, tumor-infiltrating Foxp3^+^ T_reg_ cells also expressed IL-23R, representing a sizable fraction of total IL-23R-expressing T cells in the TME (Fig. 1e,f). Only minimal *Il23r* expression was detected in myeloid cells (Extended Data Fig. 1h). We also found *Il23r* to be expressed in purified T_reg_ cells from tumors of B16 tumor-bearing *Foxp3*^DTR-GFP^ mice, whereas those from steady-state lymph nodes (LNs) and tumor-draining LNs (tdLNs) were low in *Il23r* expression (Extended Data Fig. 1i,j).

We next compared the expression of several key mediators of T_reg_ activation and suppressive functions between IL-23R^+^ and IL-23R^–^ T_reg_ cells (Fig. 1g and Extended Data Fig. 1k). IL-23R^+^ T_reg_ cells exhibited a strongly activated phenotype marked by the expression of GITR, ICOS, PD-1, CD39, CD73, CCR8, CD44 and CD69, among others (Fig. 1g). Further analysis of bulk RNA-seq data from sorted T_reg_ cells of B16 tumors or spleens^27^ confirmed high expression of *Il23r* in tumor-infiltrating T_reg_ cells, which was accompanied by an induction of key eT_reg_ genes, such as *Pdcd1* (encoding PD-1), *Tnfrsf9* (encoding 4-1BB), *Icos* and *Lag3* (Fig. 1h). Using immunofluorescence analysis of Foxp3, CD3 and IL-23R^tdTomato^ in tdLNs or B16 tumors from IL-23R^tdTomato^ reporter mice, we found IL-23R^tdTomato^-expressing T_reg_ cells in both tdLNs (Extended Data Fig. 1l) and tumors (Fig. 1i). Taken together, we identified TAMs as the major producers of IL-23 and found that IL-23R designates a highly activated T_reg_ cell subset in the mouse TME.

### T_reg_ cells mediate the tumor-promoting functions of IL-23

**Fig. 2:**
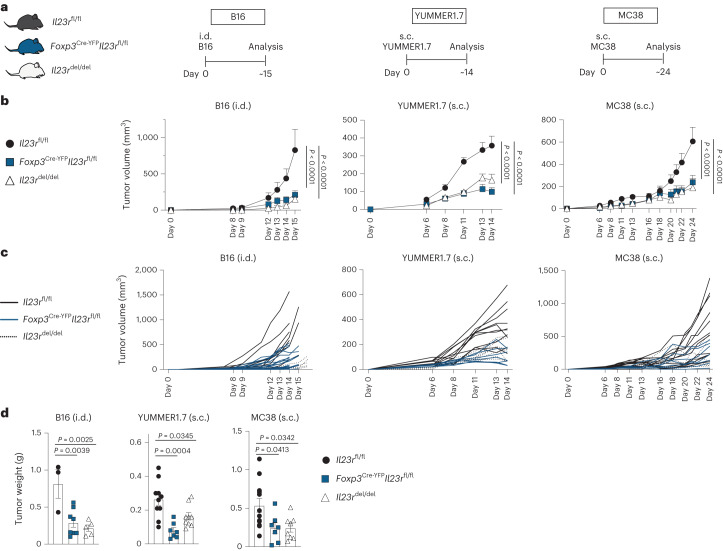
T_reg_ cells mediate the tumor-promoting functions of IL-23. **a**–**d**, *Il23r*^fl/fl^, *Foxp3*^Cre-YFP^*Il23r*^fl/fl^ and *Il23r*^del/del^ mice were inoculated i.d. with B16 tumor cells, inoculated subcutaneously (s.c.) with YUMMER1.7 tumor cells or inoculated s.c. with MC38 tumor cells, and tumors were analyzed around days 15, 14 and 24 after inoculation. The data show the results of three independent experiments (B16: *n* = 3 *Il23r*^fl/fl^ mice, *n* = 5 *Il23r*^del/del^ mice, *n* = 8 *Foxp3*^Cre-YFP^*Il23r*^fl/fl^ mice; MC38: *n* = 12 *Il23r*^fl/fl^ mice, *n* = 7 *Foxp3*^Cre-YFP^*Il23r*^fl/fl^ mice, *n* = 7 *Il23r*^del/del^ mice; YUMMER1.7: *n* = 10 *Il23r*^fl/fl^ mice, *n* = 7 *Foxp3*^Cre-YFP^*Il23r*^fl/fl^ mice, *n* = 8 *Il23r*^del/del^ mice). **a**, Schematic illustration of the experimental approach. **b**, Tumor volume kinetics of the experimental groups measured by caliper gauge. Data are shown as mean ± s.e.m. Statistical significance was determined by two-way analysis of variance (ANOVA) with a Sidak’s post hoc test. **c**, Tumor volume kinetics of individual mice measured by caliper gauge. **d**, Bar graph displaying the final tumor weight. Data are displayed as mean ± s.e.m. Statistical significance was determined using two-tailed *t*-tests. Source data

**Extended Data Fig. 2:**
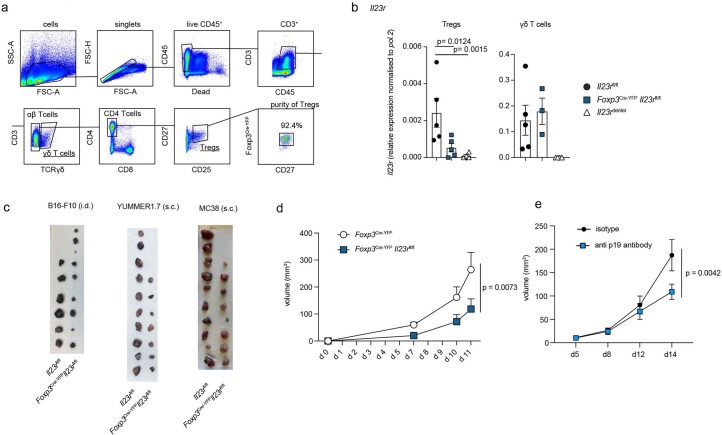
Treg cells mediate the tumor-promoting functions of IL-23. **(a**) Gating strategy to FACS sort γδ T cells and Treg cells from LNs of Foxp3^Cre-YFP^Il23r^fl/fl^ and Il23r^fl/fl^ mice for qPCR and example plot showing purity of sorted Treg cells. **(b)** Bar graphs depicting relative mRNA expression level of Il23r normalized to pol.2 in FACS sorted γδ T cells and Treg cells from steady state LNs of Foxp3^Cre-YFP^Il23r^fl/fl^, Il23r^fl/fl^ and Il23r^del/del^ mice. Data shown from one representative experiment out of two independent experiments with n = 5 (Foxp3^Cre-YFP^Il23r^fl/fl^ and IL23R^fl/fl^ mice) or n = 3 (Il23r^del/del^) biologically independent samples. Data are displayed as mean +/- SEM. Statistical significance was determined using t-tests. **(c)** Il23r^fl/fl^ and Foxp3^Cre-YFP^Il23r^fl/fl^ mice were inoculated i.d. with B16 tumor cells, inoculated s.c. with YUMMER1.7 tumor cells or inoculated s.c. with MC38 tumor cells. Tumors were analyzed on day 15, 14 or 24 post-inoculation. Pictures depict tumors after harvest. Data from 3 independent experiments with n = 6-10. **(d)** Kinetics of tumor volume measured by caliper gauge in B16 tumor cell-inoculated i.d. Foxp3^Cre-YFP^ and Foxp3^Cre-YFP^Il23r^fl/fl^ mice. Mean +/- SEM is displayed. Statistical significance was determined using 2-way Anovas. n = 4 biologically independent animals. **(e)** Kinetics of tumor volume measured by caliper gauge in MC38 tumor cell-inoculated s.c. C57Bl/6 mice i.p. injected either with anti-p19 blocking or isotype antibodies. n = 6 (isotype) and n = 7 (anti p19 antibody) biologically independent animals. Mean +/− SEM is displayed. Statistical significance was determined using 2-way Anovas. Source data

We next sought to elucidate the contribution of IL-23R signaling in T_reg_ cells to tumor progression by generating mice in which *Il23r* was specifically deleted in T_reg_ cells (*Foxp3*^Cre-YFP^*Il23r*^fl/fl^). The specificity of the conditional gene targeting was shown on T_reg_ cells, γδ T cells and CD4^+^ and CD8^+^ T cells sorted by fluorescence-activated cell sorting (FACS; Extended Data Fig. 2a,b). To model different cancer environments, we used the poorly immunogenic B16 melanoma model and the two highly infiltrated YUMMER1.7 and MC38 tumor models (Fig. 2a). We found that tumor volume and weight were drastically reduced in *Il23r*^del/del^ mice compared to in *Il23r*^fl/fl^ mice in all three tumor models (Fig. 2b–d), thus confirming the previously reported protumorigenic function of IL-23/IL-23R signaling^14–17^. Importantly, T_reg_-specific ablation of *Il23r* led to an equally reduced tumor burden, phenocopying the kinetics observed in *Il23r*^del/del^ mice, suggesting that T_reg_ cells are the relevant target of IL-23 (Fig. 2b–d and Extended Data Fig. 2c). Blockade of IL-23 with anti-p19 leads to a similar reduction in tumor growth (Extended Data Fig. 2e). To exclude the potential influence of Cre-mediated toxicity, we confirmed reduced tumor growth in the absence of *Il23r* in T_reg_ cells in *Foxp3*^Cre-YFP^ and *Foxp3*^Cre-YFP^*Il23r*^fl/fl^ mice (Extended Data Fig. 2d).

In summary, we found that T_reg_ cells mediate the tumor-promoting functions of IL-23 across different preclinical cancer models.

### IL-23R signaling in T_reg_ cells suppresses antitumor immunity

**Fig. 3:**
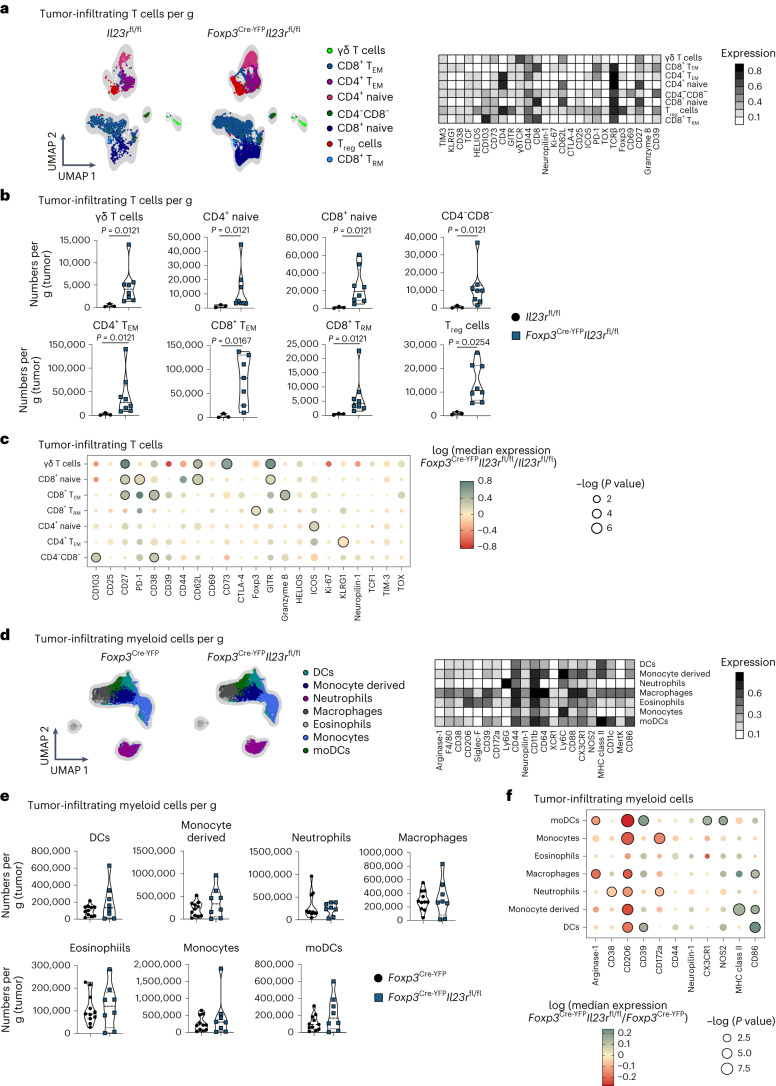
IL-23R signaling in T_reg_ cells suppresses antitumor immunity. **a**–**f**, *Il23r*^fl/fl^ and *Foxp3*^Cre-YFP^*Il23r*^fl/fl^ mice (**a**–**c**) or *Foxp3*^Cre-YFP^ and *Foxp3*^Cre-YFP^*Il23r*^fl/fl^ mice (**d**–**f**) were inoculated i.d. with B16 tumor cells, and TILs (gated on CD45^+^TCRβ^+^TCRγδ^+^ cells; **a**–**c**) or myeloid cells (gated on CD45^+^CD90.2^–^CD19^–^NK1.1^–^ cells; **d**–**f**) were analyzed by flow cytometry on day 14 after inoculation. Data are shown from one representative experiment out of two independent experiments with *n* = 3–7. **a**,**d**, UMAP with overlaid FlowSOM clustering (left) and heat map depicting relative marker expression among identified cell clusters (right). **b**,**e**, Violin plots depicting cell numbers of identified cell clusters per gram (tumor). Data are displayed as mean ± s.e.m. Statistical significance was determined using a two-tailed Mann–Whitney *U*-test. **c**,**f**, Dot plot displaying median marker expression in identified cell clusters comparing *Foxp3*^Cre-YFP^*Il23r*^fl/fl^ and *Il23r*^fl/fl^ (control group) mice (**c**) or *Foxp3*^Cre-YFP^*Il23r*^fl/fl^ and *Foxp3*^Cre-YFP^ (control group) mice (**f**). Statistical significance was determined using two-tailed *t*-tests. Color represents log (median expression *Foxp3*^Cre-YFP^*Il23r*^fl/fl^/median expression control group); that is, red indicates that median expression is decreased in *Foxp3*^Cre-YFP^*Il23r*^fl/fl^ mice compared to in the control group, and green indicates that median expression is increased in *Foxp3*^Cre-YFP^*Il23r*^fl/fl^ mice compared to in the control group. Circle size represents log (*P* value). Statistically significant changes (*P* < 0.05) are highlighted with black lines around the circles; T_RM_, resident memory T cells. Source data

**Extended Data Fig. 3:**
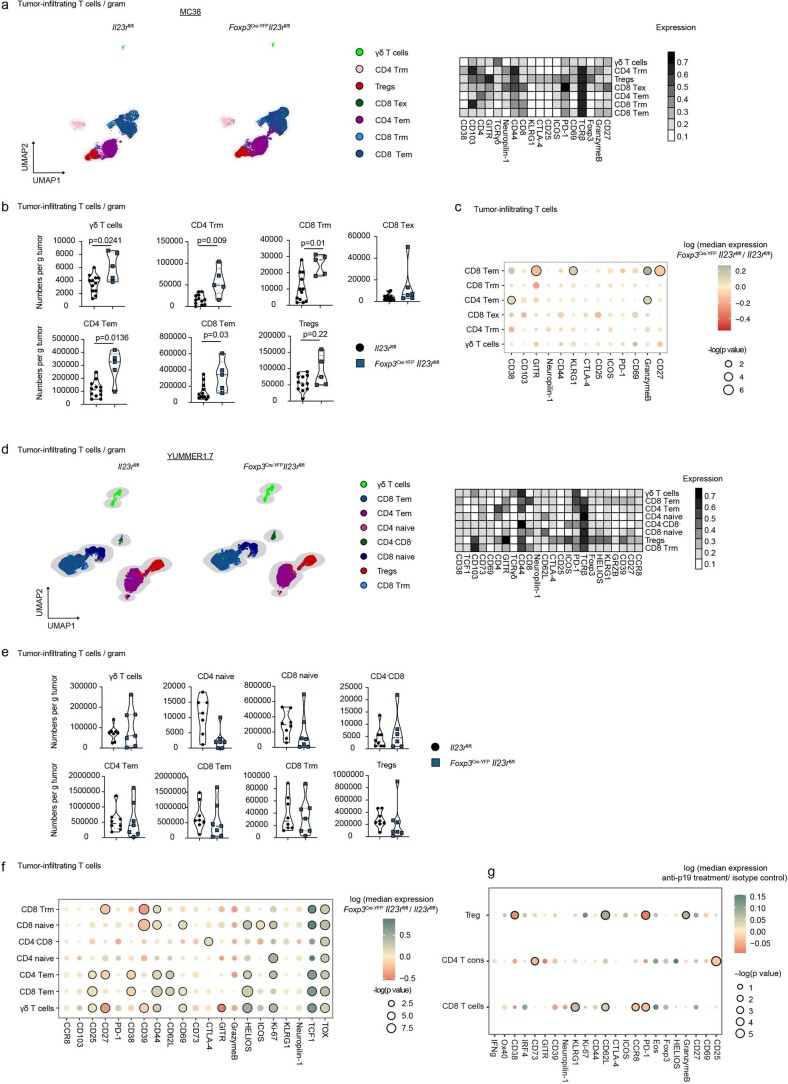
IL23R signaling in Treg cells suppresses anti-tumor immunity. **(a-f)** Il23r^fl/fl^ and Foxp3^Cre-YFP^Il23r^fl/fl^ mice were inoculated s.c. with MC38 tumor cells **(a-c)** or inoculated s.c. with YUMMER1.7 tumor cells **(d-f)** and tumor-infiltrating T cells were analyzed by flow cytometry on day 24 **(a-c)** or 14 **(d-f)** post-inoculation. TILs from MC38 tumors were re-stimulated with PMA/Ionomycin prior flow cytometry analysis. Data display 2 independent experiments with n = 6-10. **(a, d)** UMAP with overlaid FlowSOM clustering (left) (gated on CD45^+^ TCRβ^+^ and TCRγδ^+^ cells) and heatmap depicting relative marker expression among identified cell clusters (right). **(b, e)** Violin plots depicting cell numbers of identified T cell clusters per gram tumor. Data are displayed as mean +/- SEM. Statistical significance was determined using two-tailed Mann-Whitney U-tests. **(c, f)** Dotplots displaying median marker expression in identified T cell clusters comparing Foxp3^Cre-YFP^ Il23R^fl/fl^ and Il23R^fl/fl^ mice. Color represents log(median expression Foxp3^Cre-YFP^ Il23R^fl/fl^ / median expression Il23R^fl/fl^); that is red means that median expression is decreased in Foxp3^Cre-YFP^ Il23R^fl/fl^ in comparison to Il23R^fl/fl^ mice; green means that median expression is increased in Foxp3^Cre-YFP^ Il23R^fl/fl^ mice in comparison to Il23R^fl/fl^ mice. Circle size represent log(p value). Statistically significant changes (p < 0.05) are highlighted with black lines around the circles. Statistical significance was determined using t-tests. **(g)** C57Bl/6 mice were s.c. inoculated with MC38 tumor cells and tumor-infiltraring T cells were analyzed by flow cytometry on day 14 post-inoculation. Dotplot displaying median marker expression of CD4^+^T cell and Treg cell clusters comparing anti-p19 and isotype antibody treated mice. Color represents log(median expression anti-p19/median expression isotype). Circle size represent log(p value). Statistically significant changes (p < 0.05) are highlighted with black lines around the circles. Data display one out of 2 independent experiments with n = 4-7. Statistical significance was determined using t-tests. Source data

To investigate the mechanism by which IL-23R signaling in T_reg_ cells alters antitumor immunity, we profiled TILs of *Foxp3*^Cre-YFP^*Il23r*^fl/fl^ mice and *Il23r*^fl/fl^ controls. We identified eight T cell clusters including γδ T cells, T_reg_ cells and distinct differentiation stages of CD4^+^ and CD8^+^ T cells (Fig. 3a). Concomitant with the reduced tumor growth observed in *Foxp3*^Cre-YFP^*Il23r*^fl/fl^ mice, we found an increased infiltration of all T cell subsets in *Foxp3*^Cre-YFP^*Il23r*^fl/fl^ mice compared to in *Il23r*^fl/fl^ controls (Fig. 3a,b). We then included a tailored set of markers to profile activation, proliferation and dysfunction of T cells in our high-parametric single-cell phenotyping (Fig. 3a,c). Interestingly, CD8^+^ T_EM_ cells showed a more cytotoxic (granzyme B) and activated (CD38 and CD27) phenotype in tumors of *Foxp3*^Cre-YFP^*Il23r*^fl/fl^ mice (Fig. 3c). Similar observations were made in the MC38 model, where most T cell clusters and granzyme B production in CD8^+^ T_EM_ and CD4^+^ T_EM_ cells increased in *Foxp3*^Cre-YFP^*Il23r*^fl/fl^ mice (Extended Data Fig. 3a–c). Also in YUMMER1.7 tumors, TILs from mice lacking *Il23r* in T_reg_ cells, albeit not increased in number (Extended Data Fig. 3d,e), displayed a highly activated signature reflected by the expression of CD44, CD25, CD69 and Ki-67 and the transcription factors TCF-1 and TOX (Extended Data Fig. 3f), which mark CD8^+^ tumor-specific T cells transitioning toward an intermediate dysfunctional stage^28^.

Next, analyses of tumor-infiltrating myeloid cells of *Foxp3*^Cre-YFP^*Il23r*^fl/fl^ mice and *Foxp3*^Cre-YFP^ control mice identified seven distinct clusters, including macrophages, monocyte-derived cells, monocytes, monocyte-derived DCs, DCs, eosinophils and neutrophils (Fig. 3d). The numbers of myeloid subsets remained unchanged after loss of IL-23R in T_reg_ cells (Fig. 3e). However, we observed phenotypical changes in macrophages and other myeloid cells in *Foxp3*^Cre-YFP^*Il23r*^fl/fl^ mice (Fig. 3f), marked by reduced levels of arginase-1 and CD206, which are classically linked to immunosuppression and tumor progression^29–31^. By contrast, the expression of proteins enabling enhanced antigen presentation or co-stimulation, such as major histocompatibility complex class II (MHC class II) and CD86, increased (Fig. 3f). Last, we tested how neutralization of IL-23 affected TILs. Albeit less pronounced than in the genetic deletion models, we observed a more activated and less exhausted phenotype marked by higher expression of CD25 on CD4^+^ T cells and KLRG1 on CD8^+^ T cells, which featured lower PD-1 levels (Extended Data Fig. 3g). We also observed changes associated with a less suppressive signature in T_reg_ cells, denoted by decreased expression of CD38 and PD-1 (Extended Data Fig. 3g).

In summary, we found that IL-23 sensing by T_reg_ cells leads to reduced activation of antitumorigenic T effector cells and the induction of immunosuppressive features of myeloid cells.

### IL-23R signaling stabilizes eT_reg_ cells

**Extended Data Fig. 4:**
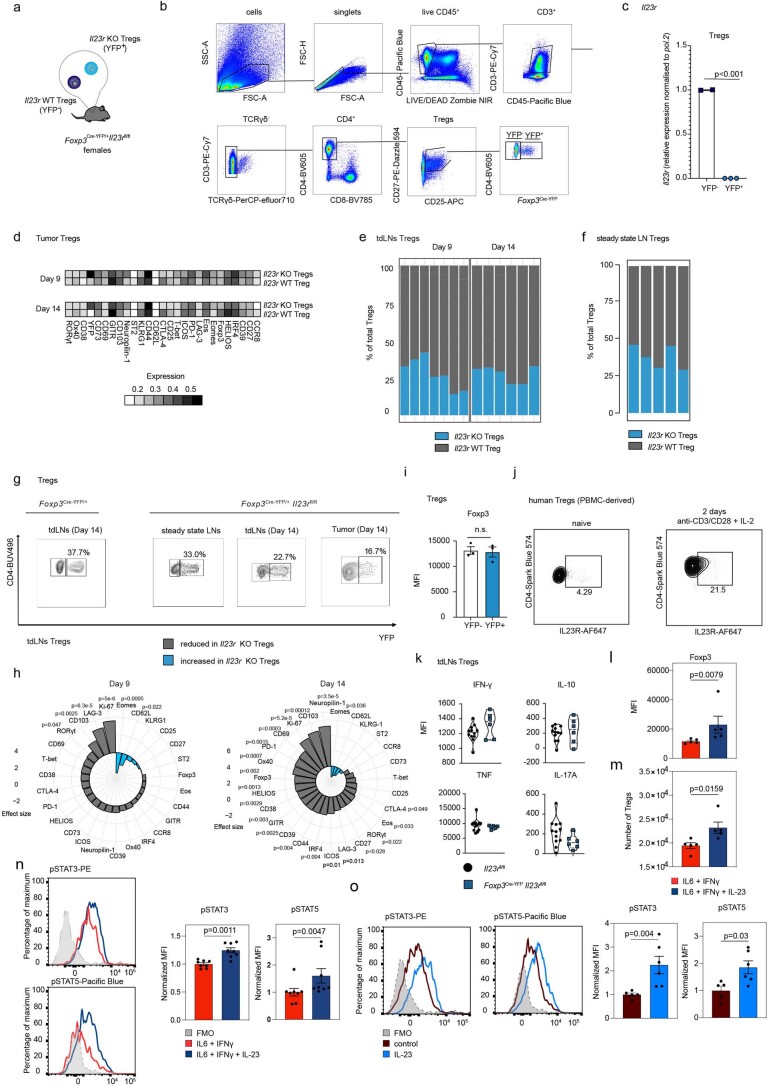
IL23R signaling confers a selective advantage on eTreg cells. **(a)** Schematic illustration of Tregs in Foxp3^Cre-YFP/+^ Il23r^fl/fl^ female mice. **(b,c**) FACS-gating strategy **(b)** and bar graphs **(c)** displaying Il23r mRNA expression levels as assessed by qPCR in YFP^+^ and YFP^−^ Treg cells from LNs of Foxp3^Cre-YFP/+^Il23r^fl/fl^ female mice. Data depict one experiment with n = 2 (YFP^-^) or n = 3 (YFP^+^). **(d-h)** Foxp3^Cre-YFP/+^Il23r^fl/fl^ female **(d,e,f,h)** and Foxp3^Cre-YFP/+^ non-floxed **(g)** mice were inoculated i.d. with B16 cells or left untreated. Treg cells in steady state LNs or tumor and tdLNs on day 9 or 14 post-inoculation were analyzed by flow cytometry. Data shown from one out of two independent experiments with n = 5-6. **(d)** Heatmap depicting marker expression among Treg cell clusters (tumor). **(e, f)** Frequency plots of Il23r KO and WT of total Treg cells in tdLNs **(e)** or steady state LNs **(f)**. **(g)** Contour plots showing YFP^+^ Treg cells on day 14. **(h)** Spiral plot displaying differential marker expression between Il23r KO and WT Treg cells (tdLNs). **(i)** Bar graph displaying Foxp3-expression in YFP^-^/YFP^+^Treg cells (tdLNs) of Foxp3^Cre-YFP/+^non-floxed mice on day 14 post-inoculation. Data from 1 experiment with n = 3. **(j)** Contour plots displaying IL23R expression as assessed by flow cytometry in human Tregs from steady state PBMCs (gated on CD45^+^ CD3^+^ CD4^+^CD25^+^CD27^+^ FOXP3^+^ cells) or 2 days anti-CD3/CD28 + IL^−^2 stimulated FACS sorted (CD45^+^CD3^+^CD4^+^CD27^+^CD25^+^CD127^−^ from steady state PBMCs) Tregs (gated on CD45^+^ CD3^+^ CD4^+^CD25^+^CD27^+^ FOXP3^+^ cells). Data shown from one experiment with n = 2. **(k)** Il23r^fl/fl^ and Foxp3^Cre-YFP^Il23r^fl/fl^ mice were inoculated i.d. with B16 cells. Treg cells in the tdLNs were analyzed by flow cytometry on day 14 post-inoculation. Violin plots displaying median expression (MFI) of cytokines. Data from one experiment with n = 6-12. **(l,m,n)** Murine Treg cells were ex vivo stimulated with IFN-γ + IL-6, + /- IL-23 for 5 days and analysed by flow cytometry. Boxplots showing the MFI of Foxp3 **(l)** and total cell numbers **(m)**. **(l,m)** n = 5.**(n**) Histograms (left) and boxplots showing normalized MFIs of pSTAT3/pSTAT5 (right). Combined result from two independent experiments with n = 8. **(o)** Murine Treg cells were ex vivo stimulated with anti-CD3/CD28 + IL-2 for 5 days and +/-IL-23 for 30 min. Histograms (left) and boxplots showing normalized MFIs (flow cytometry). Representative result from two independent experiments with n = 5 (control) or n = 6 (IL-23). **(c,i,k,l,n,o)** Data are displayed as mean +/- SEM. **(c,h,i,k,l,n,o)** Statistical significance was determined using, two-tailed t-test **(c,h)**, t-tests **(i,k,l)** or the two-tailed Mann-Whitney U-test **(n,o)**. Source data

To elucidate the mechanism by which IL-23R signaling in T_reg_ cells enhances immunosuppression, we generated *Foxp3*^Cre-YFP/+^*Il23r*^fl/fl^ female mice. In these animals, IL-23R*-*competent (wild-type (WT)) and IL-23R*-*deficient T_reg_ cells (knockout (KO)) coexist due to stochastic X chromosome inactivation^32^, resulting in a mosaic-like Cre expression, where yellow fluorescent protein (YFP) expression labels *Il23r-*ablated cells (Extended Data Fig. 4a). This was confirmed by quantitative PCR of *Il23r* expression in T_reg_ cells (Extended Data Fig. 4b,c).

**Fig. 4:**
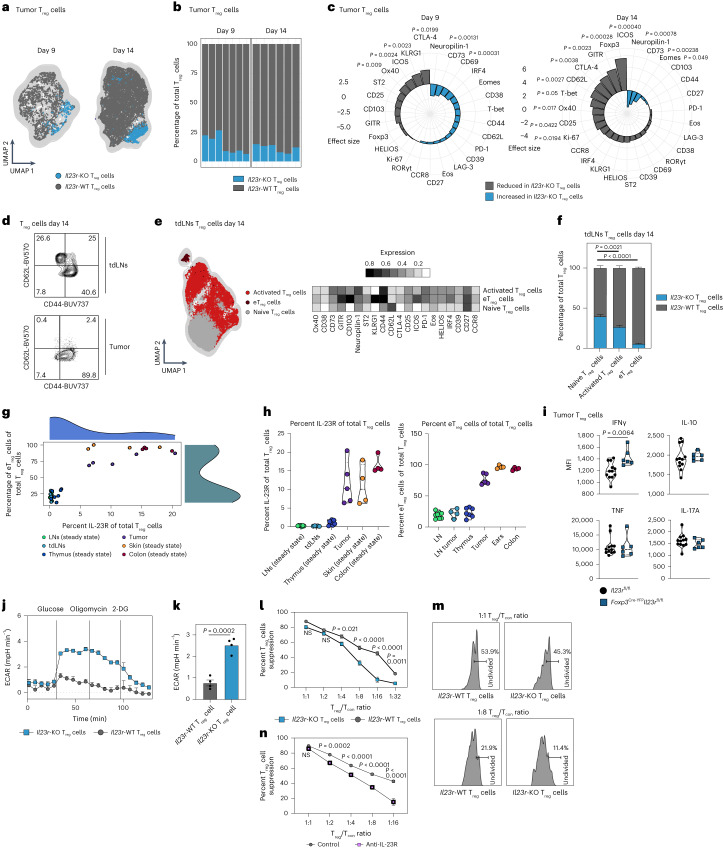
IL-23R signaling confers a selective advantage on eT_reg_ cells. **a**–**f**, *Foxp3*^Cre-YFP/+^*Il23r*^fl/fl^ female mice were inoculated i.d. with B16 cells and T_reg_ cells from tumors, and tdLNs were analyzed by flow cytometry on day 9 or 14 after injection. Data are shown from one representative experiment out of two independent experiments with *n* = 5–6. **a**, UMAP and FlowSOM clustering displaying T_reg_ cell subsets in tumors. **b**, Frequency plots of *Il23r-*KO and WT T_reg_ cells out of total T_reg_ cells in tumors. **c**, Spiral plot displaying the effect size of differential marker expression between *Il23r-*KO and WT T_reg_ cells in tumors. **d**, Representative contour plots depicting T_reg_ cells on day 14 after tumor inoculation. **e**, UMAP and FlowSOM clustering displaying T_reg_ cells in tdLNs on day 14 after tumor inoculation (left). A heat map depicting relative marker expression is shown on the right. **f**, Frequency plots of subsets of *Il23r-*KO and WT T_reg_ cells in tdLNs on day 14 after tumor injection. Statistical significance was determined by two-way ANOVA with a Sidak’s post hoc test. **g**,**h**, Scatter plot (**g**) and violin plots (**h**) displaying frequencies of IL-23R^+^ T_reg_ and eT_reg_ cells among total T_reg_ cells. Data are pooled from one to two experiments with *n* = 3–5. **i**, *Il23r*^fl/fl^ and *Foxp3*^Cre-YFP^*Il23r*^fl/fl^ mice were inoculated i.d. with MC38 tumor cells. T_reg_ cells were analyzed by flow cytometry on day 14 after inoculation. Violin plots display the median expression (median fluorescence intensity (MFI)) of cytokines. The data shown are from one experiment with *n* = 6–12. **j**, Extracellular acidification rate (ECAR) measurement of T_reg_ cells from spleens and LNs of *Foxp3*^Cre-YFP^*Il23r*^fl/fl^ or *Foxp3*^Cre-YFP^mice under the specified conditions after stimulation with anti-CD3/anti-CD28, IL-2 and IL-23 for 72 h. Data are representative of the results of two independent experiments with *n* = 4. **k**, Quantification of glycolysis in *Il23r*-KO and WT T_reg_ cells. **l**–**n**, Ex vivo suppression of CellTrace Violet-labeled CD4^+^ conventional T (T_con_) cell proliferation by *Il23r*-KO and WT T_reg_ cells (**l** and **m**) or WT T_reg_ cells ± anti-IL-23R (**n**). Data shown are from two independent experiments with *n* = 5. Statistical significance was assessed by two-way ANOVA with a Sidak’s post hoc test. Data in **f**, **i**, **k**, **l** and **n** are displayed as mean ± s.e.m. Statistical significance in **c** and **i**–**k** was determined using two-tailed *t*-tests; 2-DG, 2-deoxyglucose; NS, not significant. Source data

To explore the dynamics of *Il23r*-KO and WT T_reg_ cells, we analyzed tumor-infiltrating and tdLN-derived T_reg_ cells on days 9 and 14 after B16 tumor inoculation (Fig. 4a–f and Extended Data Fig. 4d–h). We found that *Il23r*-KO and WT T_reg_ cells clustered separately (Fig. 4a and Extended Data Fig. 4d). *Il23r*-KO T_reg_ cells accounted for less than 20% of total T_reg_ cells in tumors (Fig. 4a,b) and less than 30% in tdLNs (Extended Data Fig. 4e) compared to approximately 35% in steady-state LNs and 40% in *Foxp3*^Cre-YFP/+^ control mice (Extended Data Fig. 4f,g). This shows that, especially within the TME, *Il23r*-KO T_reg_ cells compete poorly for niche space compared to *Il23r*-WT T_reg_ cells. On day 9 after tumor inoculation, we observed reduced expression of CTLA-4, KLRG1 and ICOS in *Il23r-*KO T_reg_ cells (Fig. 4c). On day 14, the differences between *Il23r*-KO and WT T_reg_ cells further increased, as we found drastically reduced expression of key functional T_reg_ cell markers (CTLA-4, ICOS, PD-1, CD25 and GITR) as well as tissue homing factors, such as CD62L and CCR8, in *Il23r*-KO T_reg_ cells (Fig. 4c). Further, *Il23r*-KO T_reg_ cells in tumors (Fig. 4c) or tdLNs (Extended Data Fig. 4h) displayed a marked reduction of Foxp3 expression, suggesting loss of T_reg_ stability. Diminished Foxp3 expression was preceded by a reduction in expression of KLRG1 (a surrogate marker for Blimp-1 reported to stabilize Foxp3 expression^33^) on day 9 (Fig. 4c). Foxp3 expression was equal in YFP^+^ and YFP^–^ T_reg_ cells in female *Foxp3*^Cre-YFP/+^ control mice (Extended Data Fig. 4i), ruling out an artifactual Cre-mediated effect.

In agreement with previous studies^3^, the vast majority of intratumoral T_reg_ cells displayed an eT_reg_ phenotype (CD44^+^CD62L^–^; Fig. 4d). By contrast, in the tdLNs, we identified three T_reg_ differentiation stages, including activated T_reg_ cells, eT_reg_ cells and naive T_reg_ cells (Fig. 4d,e). Although the frequencies of *Il23r*-KO and WT T_reg_ cells were almost equal across naive T_reg_ cells (approximately 40% *Il23r* KO and 60% WT), the proportion of *Il23r*-KO T_reg_ cells within the activated and mostly within the eT_reg_ cluster was drastically diminished (approximately 5% *Il23r* KO and 95% WT; Fig. 4f). This suggests that eT_reg_ cells in particular depend on IL-23 sensing, which potentially explains the overall lower percentages of *Il23r*-KO T_reg_ cells in tumors where eT_reg_ cells are the dominant T_reg_ cell subtype^3,4^ (Fig. 4b and Extended Data Fig. 4e). As eT_reg_ cells are also prominent in healthy non-lymphoid tissues, we analyzed the levels of IL-23R expression and percentages of eT_reg_ cells of total T_reg_ cells across different organs using *Foxp3*^DTR-GFP^IL-23R^tdTomato^ mice (Fig. 4g,h). We found that high IL-23R expression and high percentages of eT_reg_ cells coincided in tumors and non-lymphoid tissues (steady-state skin and colon), whereas low expression of IL-23R was associated with fewer eT_reg_ cells in lymphoid tissues (steady-state LNs, tdLNs and the thymus; Fig. 4g,h), further supporting that IL-23 sensing induces or maintains eT_reg_ cells in the TME. Also, activation of human T_reg_ cells by polyclonal in vitro TCR stimulation induced IL-23R expression (Extended Data Fig. 4j).

We then analyzed the expression of effector cytokines (interferon-γ (IFNγ), IL-10, tumor necrosis factor (TNF) and IL-17A) in T_reg_ cells derived from tumors or tdLNs of *Foxp3*^Cre-YFP^*Il23r*^fl/fl^ and *Il23r*^fl/fl^ mice (Fig. 4i and Extended Data Fig. 4k). IL-17A levels remained unchanged in the absence of IL-23 sensing, but IL-23R-deficient T_reg_ cells showed enhanced expression of IFNγ in tumors but not in tdLNs (Fig. 4i and Extended Data Fig. 4k), which may actively contribute to enhanced antitumor immunity. Also, when we cultured T_reg_ cells under inflammatory conditions (IL-6 and IFNγ), IL-23 stimulation stabilized the expression of Foxp3 and the expansion of total T_reg_ cells, similar to our observations in vivo (Extended Data Fig. 4l,m). IL-23 stimulation led to enhanced phosphorylation of STAT3 and STAT5 (Extended Data Fig. 4n), which was also observed when T_reg_ cells were cultured with IL-2 for 5 d and stimulated with IL-23 for 30 min (Extended Data Fig. 4o). Also, *Il23r*-KO T_reg_ cells isolated from lymphoid tissues displayed increased glycolytic rates compared to *Il23r*-WT T_reg_ cells (Fig. 4j,k), which has been ascribed to T_reg_ cell instability^34–36^. Of note, we found that *Il23r-*KO T_reg_ cells have significantly reduced suppressive capacity compared to their WT counterparts (Fig. 4l,m), and antibody-mediated blockade of IL-23R reduced (albeit less pronounced) the suppressive capacity of T_reg_ cells (Fig. 4n).

Together, our data indicate that IL-23 confers a selective advantage for eT_reg_ cells and is crucial for T_reg_ stability and suppressive functions.

### IL-23R sensing initiates an eT_reg_ program in the murine TME

**Fig. 5:**
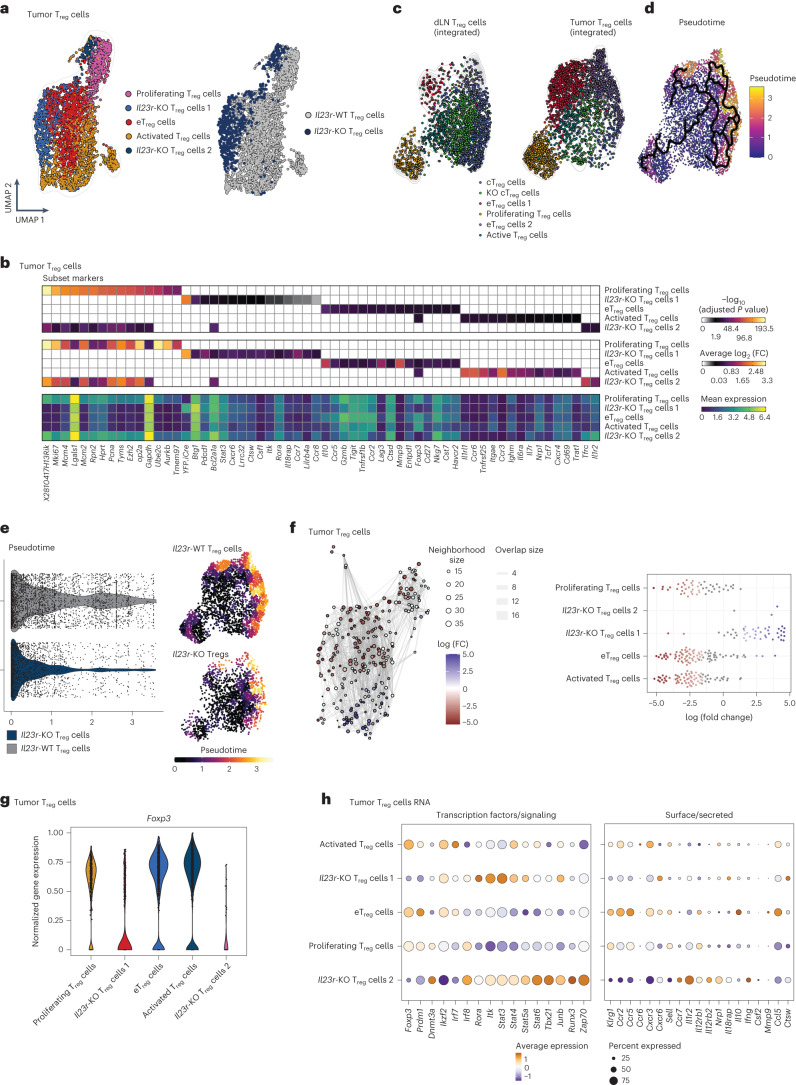
IL-23 sensing by T_reg_ cells initiates an eT_reg_ cell program in the murine TME. **a**–**h**, *Foxp3*^Cre-YFP/+^ (heterozygous) *Il23r*^fl/fl^ female mice were inoculated i.d. with B16 tumor cells, and a combined transcriptome (scRNA-seq) and protein expression analysis of sorted CD4^+^ T cells was performed on day 13 after inoculation. Data are shown from one experiment with *n* = 6. **a**, UMAP displaying identified tumor-infiltrating T_reg_ cell clusters (transcriptome; left) and UMAP highlighting *Il23*-KO and WT T_reg_ cells (right). **b**, Heat maps showing adjusted *P* value (top), average log_2_ (fold change) (log_2_ (FC); middle) and mean expression (bottom) of subset markers in the identified T_reg_ cell clusters assessed by Wilcoxon rank-sum test and Benjamini–Hochberg correction. The complete list is available in Supplementary Table 1. **c**, UMAP displaying identified clusters of integrated tumor-infiltrating and tdLN-derived T_reg_ cells. **d**, UMAP of tdLN and tumor T_reg_ cells with overlayed pseudotime and principal graph lines calculated with Monocle 3. **e**, Violin plot (left) and UMAP (right) comparing the distribution of WT and *Il23r*-KO T_reg_ cells along pseudotime (corresponding to **d**). **f**, Neighborhood graph of DA testing results (left). Coloring indicates log (fold change) of differentially abundant neighborhoods between WT and *Il23r*-KO T_reg_ cells. White neighborhoods are not differentially abundant (false discovery rate of 10%). Dot size corresponds to the number of cells per neighborhood, and edges indicate the number of overlapping cells between neighborhoods. The index cell position in UMAP space (**a**) determines ordering of the neighborhood nodes. The Beeswarm plot (right) indicates the distribution of differentially abundant neighborhoods across clustering-based T_reg_ cell subsets. **g**, Violin plots depicting the normalized *Foxp3* RNA abundance among identified T_reg_ cell subsets. **h**, Dot plot displaying selected DEGs between identified T_reg_ cell subsets encoding transcription factors/signaling proteins (left) and surface or secreted proteins (right). The complete list is available in Supplementary Table 2; cT_reg_, central T_reg_ cells.

**Extended Data Fig. 5:**
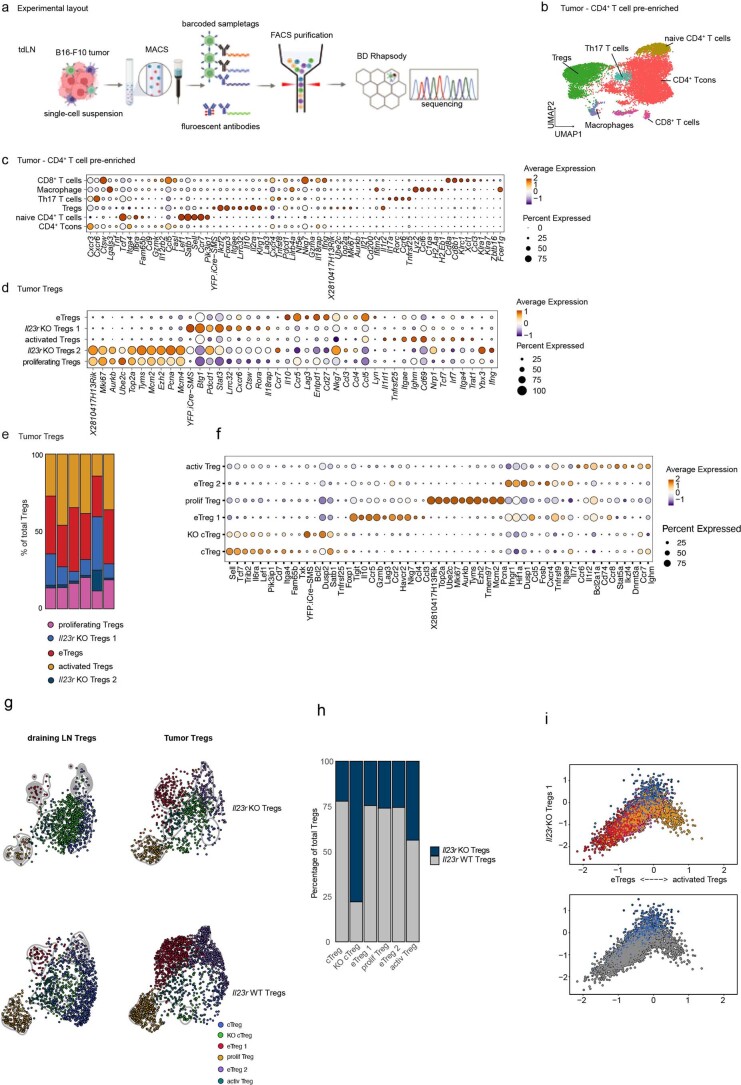
IL-23 sensing by Treg cells initiates an eTreg cell program in the murine TME. **(a-i)** Foxp3^Cre-YFP/+^ (heterozygous) Il23r^fl/fl^ female mice were inoculated i.d. with B16 tumor cells and combined transcriptome (scRNAseq) and protein expression analysis of sorted CD4^+^T cells was performed on day 13 post-inoculation. Data shown from one experiment with n = 6. **(a)** Schematic illustration of the experimental workflow. **(b)** UMAP displaying clustered (based on transcriptome expression) and manually annotated cell subsets passing quality control. **(c)** Dotplot depicting the 10 most variable features in all identified cell subsets. **(d)** Dotplot displaying the 10 most variable features across identified Treg cell subsets. **(e)** Bar chart displaying the frequencies of Treg cell subsets of total tumor Treg cells. **(f)** Dotplot of the most variable features of Treg cell subsets of integrated tumor and tumor-draining lymph node Treg cells. **(g)** UMAPs highlighting the distribution of Il23r KO and WT Treg cells across identified clusters in tumors and tumor-draining lymph nodes. **(h)** Bar chart of percentages of Il23r KO and WT Treg cells across Treg cell clusters. **(i)** Cellular state plot displaying enrichment of module scores of identified Treg cell subsets compared to gene modules of activated vs. eTreg cells (x-axis) and KO Treg cells (y-axis).

To identify the downstream effects of IL-23R signaling in T_reg_ cells, we analyzed pre-enriched CD4^+^ T cells isolated from B16 tumors and tdLNs (day 13) of *Foxp3*^Cre-YFP/+^*Il23r*^fl/fl^ female mice using targeted proteogenomic profiling, simultaneously capturing transcriptome and surface marker expression at the single-cell level (Extended Data Fig. 5a). We mapped three subsets of *Il23r*-WT T_reg_ cells (proliferating T_reg_ cells, activated T_reg_ cells and eT_reg_ cells) and two clusters of *Il23r*-KO T_reg_ cells (*Il23r*-KO T_reg_ cell 1 and *Il23r*-KO T_reg_ cell 2; Fig. 5a, Extended Data Fig. 5b–e and Supplementary Table 1). This confirmed a reduced proportion of both *Il23r*-KO T_reg_ clusters compared to the *Il23r*-WT clusters (Fig. 5a and Extended Data Fig. 5e). Furthermore, the *Il23r*-KO T_reg_ cell clusters showed a highly distinct expression signature (Fig. 5b and Extended Data Fig. 5d). We then integrated our transcriptome data to capture a broad spectrum of T_reg_ cell states that could be differentially ascribed to tumors or tdLNs (Fig. 5c and Extended Data Fig. 5f). As expected^37^, tumors were enriched for both eT_reg_ cell clusters. Conversely, tdLNs contained more central T_reg_ cells, including a KO-specific central T_reg_ cell cluster. Trajectory inference analysis confirmed that tumor-infiltrating eT_reg_ cells represented the most differentiated state (Fig. 5d). Strikingly, we found that *Il23r*-KO T_reg_ cells had a profound reduction in the proportion of eT_reg_ cells compared to WT cells (Fig. 5e and Extended Data Fig. 5g,h). Mapping the signature of intratumoral *Il23r-*KO T_reg_ cells along a gradient from activated to eT_reg_ cells further revealed that *Il23r*-KO T_reg_ cells differ from eT_reg_ cells, suggesting that the differentiation to an eT_reg_ stage requires IL-23R signaling (Extended Data Fig. 5i).

**Extended Data Fig. 6:**
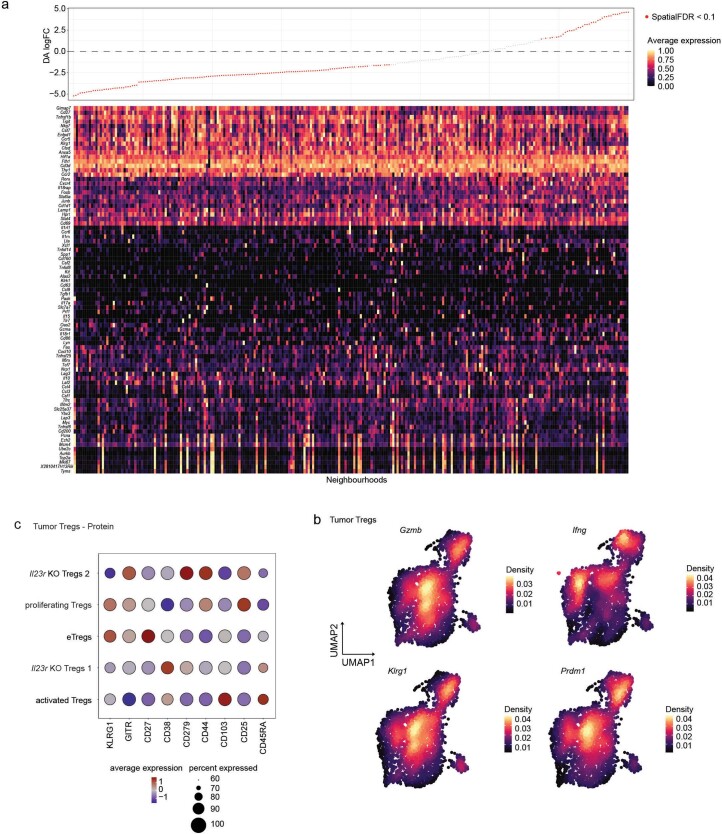
IL-23 sensing by Treg cells initiates an eTreg cell program in the murine TME. **(a-c)** Foxp3^Cre-YFP/+^ (heterozygous) Il23r^fl/fl^ female mice were inoculated i.d. with B16 tumor cells and combined transcriptome (scRNAseq) and protein expression analysis of sorted CD4^+^T cells was performed on day 13 post-inoculation. **(a)** Log fold change of DA neighborhoods marked in red, neighborhoods with spatial FDR > 0.1 marked in grey (top). Heatmap depicting average gene expression of most variable markers across neighborhoods (bottom). **(b)** Dotplot depicting differentially expressed protein markers between the identified Treg cell clusters. **(c)** Expression density of selected variable features overlayed on UMAP displaying tumor Treg cells.

Assigning single cells to partially overlapping neighborhoods on a *k*-nearest neighbor graph^38^, which allows for differential abundance (DA) testing of graph neighborhoods, revealed that WT and *Il23r*-KO T_reg_ cells indeed form distinct cellular states (Fig. 5f and Extended Data Fig. 6a). By superimposing the DA results to the single-cell embedding, we found that the mostly differing neighborhoods of *Il23r*-WT T_reg_ cells located to the eT_reg_, activated and proliferating T_reg_ cell clusters, as opposed to *Il23r*-KO T_reg_ cells, which showed showed no enrichment in these clusters (Fig. 5f and Extended Data Fig. 6a).

The evaluation of the differentially expressed genes (DEGs) between intratumoral T_reg_ cell subsets (Supplementary Table 2) confirmed a marked reduction of *Foxp3* expression in *Il23r-*KO T_reg_ cells (Fig. 5g). This was accompanied by a decrease in *Prdm1* (encoding Blimp-1) expression in both *Il23r*-KO T_reg_ cell subsets and an increase in expression of *Dnmt3a* in the *Il23r*-KO T_reg_ cell 2 subset. In addition, the *Il23r*-KO T_reg_ cell 2 subset showed increased expression of *Ifng*, *Csf2*, *Tbx21* and *Ybx3* (Fig. 5h and Extended Data Fig. 5f), previously associated with T_reg_ cell destabilization^39^. Both *Il23r*-KO T_reg_ cell clusters featured enhanced expression of *Rora*, *Stat3*, *Stat4*, *Stat5a* and *Stat6*, profound changes in the expression of genes encoding chemokine receptors (*Ccr2, Ccr5, Ccr6, Cxcr3* and *Ccr7*; Fig. 5h and Extended Data Fig. 5d) and a marked reduction in the expression of *Klrg1*, which was confirmed at the protein level (Extended Data Fig. 6b). In line with this finding, we found that the highest density of cells expressing *Prdm1*, *Klrg1* and *Gzmb* located to WT *Il23r* T_reg_ cell clusters, in contrast to *Ifng*, which showed the highest density in *Il23r*-KO T_reg_ cells (Extended Data Fig. 6c).

In summary, our data demonstrate that IL-23R signaling in mouse T_reg_ cells stabilizes their differentiation to an eT_reg_ state with enhanced function and stability.

### IL-23R signaling induces an eT_reg_ program in the human TME

**Fig. 6:**
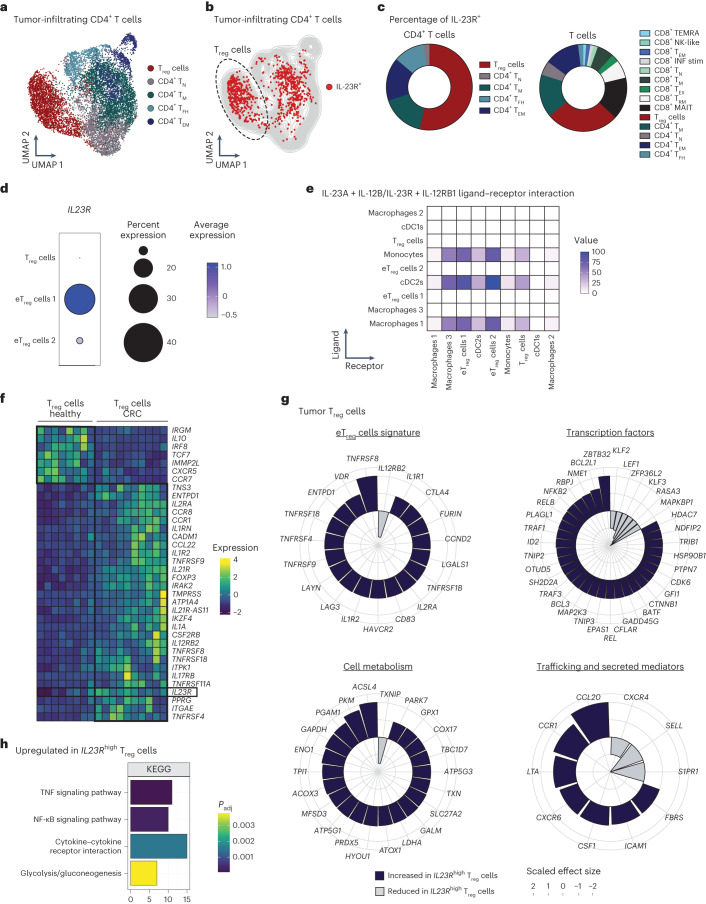
IL-23R signaling induces an eT_reg_ cell program in the human TME. **a**–**c**,**g**,**h**, Analyses of a human pan-cancer single-cell sequencing dataset^41^. **a**, UMAP displaying pan-cancer CD4^+^ T cells. **b**, UMAP highlighting *IL23R*^+^CD4^+^ T cells. **c**, Pie chart depicting the frequencies of T cell subsets among total *IL23R*^+^CD4^+^ T cells (left) and among total *IL23R*^+^ T cells (right). **d**,**e**, Analysis of an scRNA-seq dataset of human colorectal carcinomas (Liu et al.^43^). **d**, Dot plot displaying *IL23R* expression across T_reg_ cell subsets. **e**, Interaction heat map based on inferred ligand–receptor score between myeloid and T_reg_ cell subsets of the IL-23A + IL-12B/IL-23R + IL-12RB1 axis computed with ICELLNET. The intensity of communication score is depicted as color intensity value. **f**, Heat map depicting the median expression of selected genes among the top 50 DEGs between T_reg_ cells isolated from healthy colon biopsies and from tumor tissue of individuals with colorectal cancer from a bulk next-generation sequencing dataset (Magnuson et al.^27^). Expression of *IL23R* is highlighted. **g**, Spiral plots displaying the scaled (positive values between 1 and 2; negative values between −1 and −2) effect size of selected DEGs between *IL23R*^high^ (*IL23R* expression > 0) and *IL23R*^low^ (*IL23R* expression = 0) T_reg_ cells. The complete list of DEGs is available in Supplementary Table 3. **h**, Selected significantly enriched pathways from KEGG pathway analysis using G:Profiler comparing *IL23R*^high^ and *IL23R*^low^ T_reg_ cells. Significance was calculated by g:GOSt using a Fisher’s one-tailed test. No downregulated pathways were detected. The complete list is available in Supplementary Table 4; T_N_, naive T cells; T_EX_, exhausted T cells; T_M_, memory T cells; T_FH_, follicular helper T cells; MAIT, mucosal-associated invariant T cells; *P*_adj_, adjusted *P* value. Source data

**Extended Data Fig. 7:**
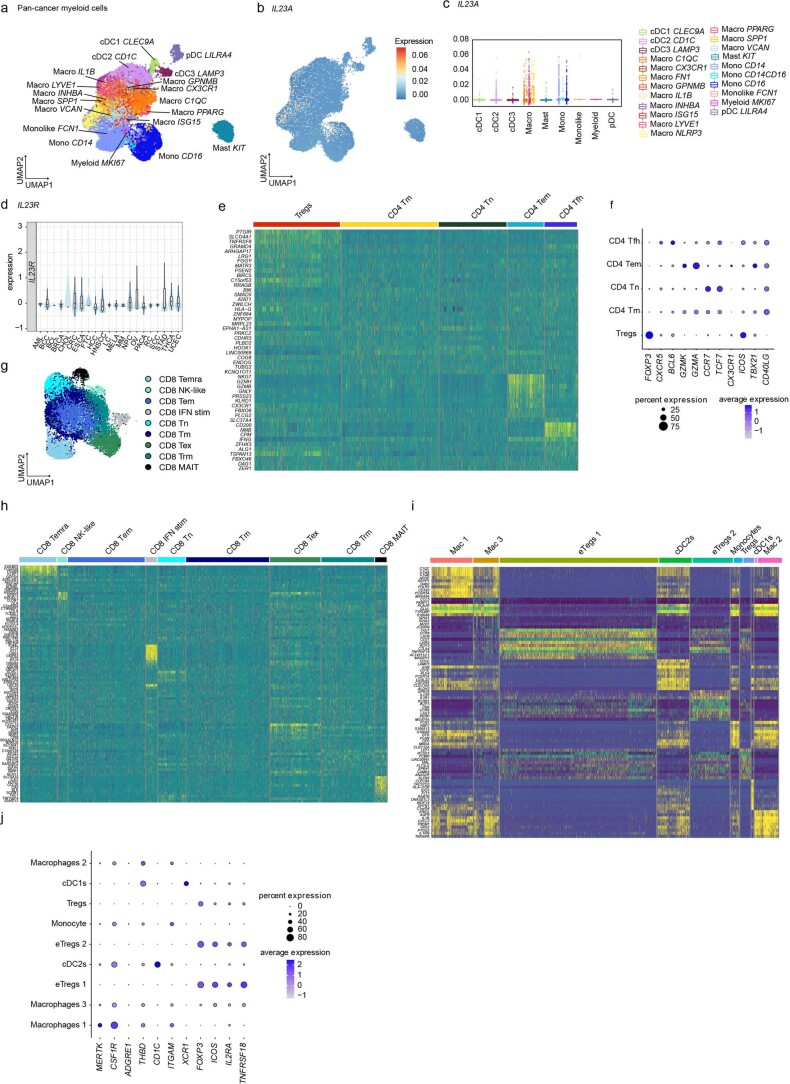
IL23R signaling induces an eTreg cell program in the human TME. **(a**–**c)** Analyses of human myeloid pan-cancer single-cell sequencing data set from (Cheng et al. 2021) from n = 210 individual patients across 15 human cancer types. **(a)** UMAP displaying pan-cancer myeloid cells. **(b)** UMAP depicting pan-cancer myeloid cells with overlaid IL23A expression. **(c)** Box plot displaying average IL23A expression in different pan-cancer myeloid cell clusters (grouped by cell type). Boxplots display the median and interquartile range (IQR; 25-75%) with whiskers representing the upper and lower quartile +/- IQR. (**d**) Box plot displaying IL23R expression among Treg cells from patients with different cancer types. Boxplots display the median and interquartile range (IQR; 25-75%) with whiskers representing the upper and lower quartile +/- IQR. **(e-i)** Analyses of human pan-cancer single-cell sequencing data set from Zheng et al.^41^ **(e, f, g, h)** and analyses of scRNAseq dataset of human colorectal carcinomas (Liu et al.^43^) **(i, j)**. **(g)** UMAP depicting pan-cancer CD8^+^ T cells. **(e, h, i)** Heatmap depicting the expression of cell-type defining genes. **(f, j)** Dotplots showing the expression of selected cell lineage markers.

**Extended Data Fig. 8:**
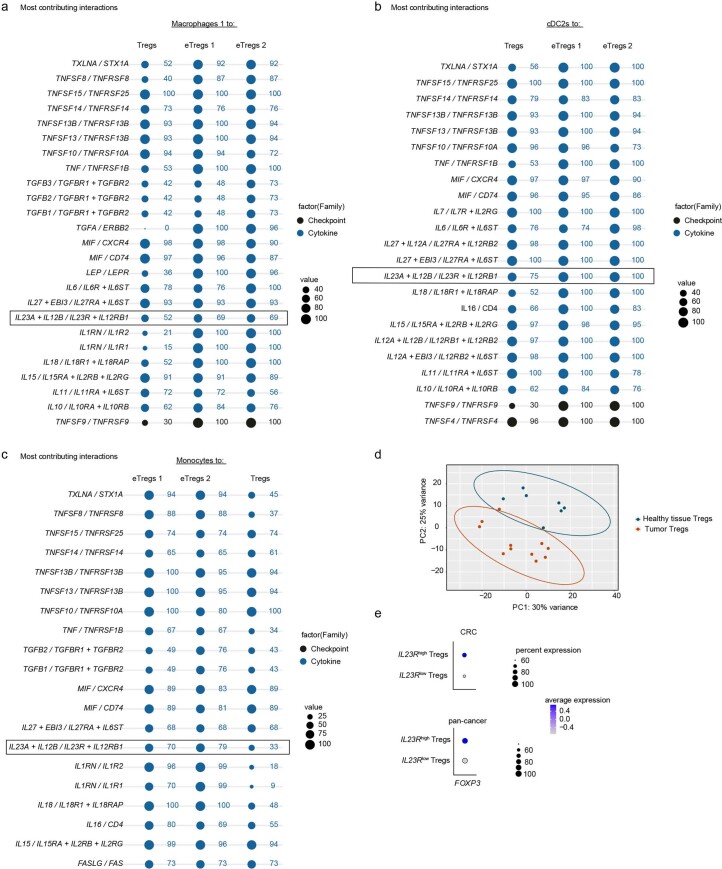
IL-23 signaling induces an eTreg cell program in the human TME. **(a**–**c)** Analyses of scRNAseq dataset of human colorectal carcinomas (Liu et al.^43^). Dotplots displaying the top 25 statistically significant outgoing interactions from the macrophages 1 **(a)**, cDC2s **(b)** or monocytes **(c)** cell clusters to Treg cell clusters computed with the ICELLNET framework. **(d)** PCA depicting data from bulk NGS data set from (Magnuson et al.^27^). Comparison between Treg cells isolated from healthy colon biopsies and tumor tissue from colorectal cancer patients. **(e)** Dotplots displaying FOXP3 gene expression in IL23R^high^ (IL23R expression > 0) and IL23R^low^(IL23R expression = 0) Treg cells from the human pan cancer T cell atlas (Zheng et al. 2021) and colorectal carcinoma (Liu et al.^43^) scRNA seq data sets.

To assess the translational value of our findings in the context of human cancer, we analyzed three bulk and scRNA-seq datasets^27,40,41^ (Fig. 6 and Extended Data Figs. 7 and 8). In agreement with our data above, we found that TAMs were the main source of *IL23A* (encoding IL-23p19) across multiple human cancer entities^41^ (Extended Data Fig. 7a–c).

To characterize *IL23R* expression in the TME at the single-cell level, we analyzed an scRNA-seq T cell atlas including 21 malignant entities from 316 individuals^41^ (Fig. 6a–c and Extended Data Fig. 7d–h). We assigned eight CD8^+^ and five CD4^+^ T cell clusters including a T_reg_ cell cluster (Fig. 6a and Extended Data Fig. 7e–h). Several CD4^+^ T cell clusters expressed *IL23R*, including CD4^+^ memory T cells, T_EM_ cells and follicular helper T cells (Fig. 6b,c), whereas CD8^+^ mucosal-associated invariant T cells, CD8^+^ resident memory T cells and CD8^+^ memory T cells were identified as the main *IL23R*-expressing CD8^+^ T cell clusters (Fig. 6c). T_reg_ cells represented the main *IL23R*-expressing cancer T cell cluster, accounting for over 50% of the *IL23R*^*+*^CD4^+^ T cells (Fig. 6b,c) and 29% of total *IL23R*^+^ pan-cancer T cells (Fig. 6c). *IL23R*-expressing T_reg_ cells could be identified across a wide variety of human cancer entities (Extended Data Fig. 7d).

To discern the cross-talk between T_reg_ cells and myeloid cells within the TME, we performed cell–cell communication analysis^42^ on an scRNA-seq dataset of colorectal cancer tissue^43^ (Fig. 6d,e and Extended Data Fig. 7i,j). Among the three identified T_reg_ cell clusters, two displayed an eT_reg_ gene signature and higher expression of *IL23R* than the less activated T_reg_ cell cluster resembling that of peripheral blood T_reg_ cells (Fig. 6d)^43^. Among myeloid cells, we identified cDC1s, cDC2s, monocytes and three macrophage subsets^44,45^. Cell–cell communication analysis revealed highly predicted ligand–receptor interactions via the IL-23A + IL-12B/IL-23R + IL-12RB1 axis between myeloid cells (macrophage cluster 1, monocytes and cDC2s) and eT_reg_ cells (Fig. 6e), which was among the top 25 predicted interactions (Extended Data Fig. 8a–c).

In bulk RNA-seq data comparing T_reg_ cells from healthy colon biopsies and tumor tissue from individuals with colorectal cancer^27^ (Fig. 6f and Extended Data Fig. 8d), we found elevated *IL23R* expression in tumor T_reg_ cells, which coincided with increased expression of *FOXP3* and its target genes *IL2RA*, *IKZF4*, *ENTPD1* (encoding CD39) and *TNFRSF18* (encoding GITR; Fig. 6f).

To pinpoint how IL-23R signaling shapes T_reg_ cells at the single-cell level, we next compared the gene expression profile of *IL23R*^high^ and *IL23R*^low^ pan-cancer T_reg_ cells from 21 different entities^41^ (Fig. 6g,h). We found 216 DEGs (Supplementary Table 3), which we categorized into genes related to the ‘eT_reg_ signature’, ‘transcription factors’, ‘cell metabolism’ and ‘T_reg_ cell trafficking and secreted mediators’ (Fig. 6g and Supplementary Table 3). We additionally identified IL-23-induced pathways in T_reg_ cells^46,47^ (Fig. 6h and Supplementary Table 4). *IL23R*^high^ T_reg_ cells showed an eT_reg_ gene expression profile^48,49^ marked by higher expression of *TNFRSF8* (encoding CD30), *VDR*, *ENTPD1* (encoding CD39), *TNFRSF18* (encoding GITR), *TNFRSF4* (encoding OX-40), *TNFRSF9* (encoding 41-BB), *LAYN*, *LAG3*, *IL1RN*, *IL1R2*, *HAVCR2* (encoding TIM-3), *IL2RA* (encoding CD25), *CTLA-4* and others (Fig. 6g). In line with the increased expression of several TNFRSFs and cytokine receptors, TNF signaling and cytokine/cytokine receptor interaction pathways were enriched in *IL23R*^high^ T_reg_ cells (Fig. 6h). Of note, the expression of *IL12RB2* was reduced in *IL23R*^high^ T_reg_ cells (Fig. 6g).

IL-23-sensing T_reg_ cells showed marked expression of *BATF*, *PLAGL1*, *SH2D2A*, *ZFP36L2*, *NDFIP2* and *CFLAR* (Fig. 6g)^50^. Also, the NF-κB signaling pathway (Fig. 6h) and its associated genes, including *NFKB2* and *REL* (encoding Rel-c), as well as genes encoding trafficking and secreted molecules (*CCR1*, *CXCR6*, *ICAM-1*, *S1PR1*, *SELL* (encoding l-selectin), *CXCR4*, *CCL20*, *LTA*, *CSF1* and *FBRS*) were elevated in expression in *IL23R*^high^ T_reg_ cells (Fig. 6g).

Overarchingly, we observed a trend toward higher *FOXP3* expression in *IL23R*^high^ T_reg_ cells in both the pan-cancer T cell atlas and in a separate single-cell transcriptome dataset from individuals with colorectal cancer^43^ (Extended Data Fig. 8e). *IL23R*^high^ T_reg_ cells also displayed a distinct metabolic profile (Fig. 6g).

Together, these data indicate that IL-23R signaling in T_reg_ cells is a prominent feature across human cancers, promoting a highly suppressive eT_reg_ cell signature^49^.

## Discussion

Here, we identified a crucial role for IL-23R signaling in stabilizing an effector T_reg_ cell program in the TME. Thus far, identifying IL-23R-expressing cells has been challenging due to the poor specificity of available antibodies and low expression of IL-23R. Nevertheless, two reports have suggested that T_reg_ cells express IL-23R in preclinical models of cancer^14,22^. By generating an IL-23R reporter mouse, we demonstrated IL-23R expression in T_reg_ cells in the TME. Although we found that other T cell subsets express IL-23R, specific ablation of *Il23r* in T_reg_ cells resulted in reduced tumor growth, phenocopying full *Il23r*-KO mice.

TAMs are the predominant IL-23 source in the mouse and human TME, and ligand–receptor interaction analyses consequently indicated potential interactions between these myeloid cells and T_reg_ cells. It is well established that T_reg_ cells mediate some of their suppressive functions via antigen-presenting cells through a variety of mechanisms, including the theft of CD80/CD86 via CTLA-4 (refs. ^51,52^), the depletion of MHC class II^53^ or the reprogramming of macrophages toward an anti-inflammatory phenotype^54^. Along this line, we found an increase in CD86 and MHC class II and a decrease in CD206 in several myeloid subsets from tumors where T_reg_ cells cannot sense IL-23. We thus propose that likely not only one but several mechanisms may be responsible for the myeloid cell reprogramming observed in *Foxp3*^Cre-YFP^*Il23r*^fl/fl^ mice.

In line with previous observations^14,15,17^, we found an expansion of effector T cells and T_reg_ cells in the T_reg_ cell-specific *Il23r*-KO mouse strain in two tumor models. Of note, the activation status of (non-T_reg_) TILs showed some model-specific variations, which may be explained by differences in the degree of immunogenicity, growth kinetics and time points of analysis. Nonetheless, the overall phenotype, including enhanced activation and effector function, was maintained across all models analyzed. Some differences observed when comparing tumors from *Foxp3*^Cre-YFP^*Il23r*^fl/fl^ and control mice might stem from the distinctive inflammatory milieus found in tumors of vastly different sizes. We circumvented this problem using female *Foxp3*^Cre-YFP/+^*Il23r*^fl/fl^ mice, which allowed us to compare *Il23r*-KO and WT T_reg_ cells within the same tumor. When coexisting in the same TME, *Il23r*-KO T_reg_ cells displayed reduced expression of Foxp3 compared to WT T_reg_ cells, which was preceded by a decrease in the expression of KLRG1, a surrogate marker for Blimp-1. We hypothesize that Blimp-1 can be induced by IL-23, preventing Foxp3 methylation and thus stabilizing T_reg_ cell identity within the TME^33^. Indeed, *Il23r*-KO T_reg_ cells exhibited a reduction in the expression of *Prdm1* (encoding Blimp-1) and an increase in the expression of *Dntm3a*, which has been shown to methylate Foxp3 at the CNS2 region, thereby contributing to T_reg_ cell destabilization^55^. Furthermore, the genetic signature of Blimp-1-competent compared to Blimp-1-deficient T_reg_ cells is reminiscent of the impact of IL-23 on T_reg_ cells^56^.

Further indicative of their instability, T_reg_ cells lacking *Il23r* showed halted differentiation to late eT_reg_ cell stages and a high expression of IFNγ, which is usually suppressed via the Foxp3–Runx1 axis in T_reg_ cells^57^. Of note, IFNγ production by T_reg_ cells has recently been associated with a defective eT_reg_ cell program^58^, and IL-23R-deficient T_reg_ cells may directly promote antitumor responses via enhanced IFNγ production. Further supporting the notion that IL-23 stabilizes T_reg_ cells, we found increased glycolysis rates in T_reg_ cells lacking IL-23R. Unconstrained glycolysis, which can be limited by Foxp3, contributes to T_reg_ cell destabilization^35^. This is consistent with a recent report showing reduced tumor growth in *Foxp3*^Cre-YFP^*Il23r*^fl/fl^ mice using the MC38 tumor model^22^. Wight et al. observed increased *Il12rb2* (but not *Il12rb1*) transcripts and IL-12RB2 protein expression in *Il23r*-KO T_reg_ cells and proposed that lack of *Il23r* in T_reg_ cells might enhance their sensitivity for IL-12 signaling by increasing the availability of *Il12rb1* to form the IL-12 receptor with *Il12rb2* (ref. ^22^). Although we also observed increased expression of *Il12rb2* transcripts in *Il23r*-KO T_reg_ cells, *Il12rb1* transcript expression remained unchanged or was slightly elevated, indicating that enhanced IL-12 responsiveness might be one but not the only mechanism explaining the phenotype of *Il23r*-KO T_reg_ cells. Overall, our data suggest that IL-23R transmits a fundamental signal to promote eT_reg_ cell function involving Foxp3 and its downstream targets and therefore does not only serve as a decoy mechanism to prevent the formation of the IL-12 receptor. Because in vitro TCR stimulation of T_reg_ cells was sufficient to induce IL-23R expression, we presume that T_reg_ cells, after antigen encounter in the TME, undergo initial activation of IL-23R expression. In turn, IL-23 sensing allows the stabilization of the full eT_reg_ phenotype and local suppression of antitumor immunity. Interestingly, *Il23r* was among the most upregulated genes in T_reg_ cells after loss of Blimp-1 (ref. ^56^), suggesting a feedback loop that might also explain why only a fraction of eT_reg_ cells expressed IL-23R. Importantly, we could translate our preclinical findings to human cancer. We found that *IL23R*^high^ T_reg_ cells derived from human tumors are marked by higher expression of key genes encoding eT_reg_ cell molecules.

This report reveals an unexpected immunosuppressive property of an otherwise proinflammatory cytokine. In hindsight, it is not surprising that mediators that can cause immunopathology also engage with regulatory elements such as T_reg_ cells to limit tissue destruction and terminate immune responses. We can speculate that this dichotomy is inherent across other proinflammatory mediators. In the case of IL-1β, this has recently been proposed^59^. Here, this unexpected role of IL-23R signaling in stabilizing T_reg_ suppressive functions sets the sound base for the therapeutic targeting of T_reg_ cells through IL-23 or IL-23R blockade to expand the armamentarium of cancer immunotherapy.

## Methods

### Mice

B6.129(Cg)-*Foxp3*^*tm3*^^(DTR/GFP)Ayr^/J (*Foxp3*^DTR-GFP^) mice and B6.129(Cg)-*Foxp3*^*tm4*(YFP/icre)Ayr^/J (*Foxp3*^Cre-YFP^) mice were purchased from The Jackson Laboratory (016958 and 016959, respectively). *Il23r*^fl/fl^ mice were obtained from P. Rosenstiel, University of Kiel, Germany^60^. *Il23r*^fl/fl^ mice were crossed to a Deleter-Cre line CMV (Deleter) Cre (006054) to obtain *Il23r*^del/del^ mice. IL-23R^tdTomato^ mice were generated by M. Oukka, Children’s Hospital Seattle, USA and Biocytogen plasmid construction service. Mice were maintained on a C57BL/6 background and were housed in a specific pathogen-free environment. Both female and male mice were used for experiments at the age of 6–10 weeks. Mice were socially housed with a dark/light cycle of 12 h, ambient temperature of 22 °C and 45–65% humidity. All experiments were approved by the Cantonal Veterinary Office of Zurich.

### Mouse tumor models

B16 cells were originally received from Xenogen. The MC38 cell line was received from M. Dettmer, ETH Zurich, Switzerland. The YUMMER1.7 cell line was purchased from Merck-Millipore. Mice were inoculated i.d. with 1.5 × 10^5^ B16 cells, s.c. with 2 × 10^6^ YUMMER1.7 cells or s.c. with 3 × 10^5^ MC38 cells. Starting from day 7 after injection, tumor size and body weight were measured. Measurements were first performed three times a week and later daily. Mice were killed by CO_2_ inhalation.

### Tissue processing

Tumors were minced into pieces and digested in RPMI supplemented with 2% fetal calf serum (FCS), 1 mg ml^–1^ collagenase IV and 100 µg ml^–1^ DNase I (both Sigma-Aldrich) at 37 °C for 45 min. Tissues were then disrupted with a syringe (18-gauge needle) and digested for another 15 min. Cells were then filtered through 100-µm cell strainers and washed with PBS. LNs and thymi were ground through 100-µm cell strainers and washed with PBS. Immune cells were enriched using mouse CD45 TIL microbeads (Miltenyi Biotec) following the manufacturer’s instructions.

To digest ear skin, skin was minced into pieces and digested in RPMI supplemented with 2% FCS, 1 mg ml^–1^ collagenase IV and 100 µg ml^–1^ DNase I (both Sigma-Aldrich) at 37 °C for 1.5 h (ref. ^61^). Skin tissue was disrupted with a syringe (18-gauge needle) and filtered through 70-μm cell strainers.

To isolate immune cells from mouse colons, 6-cm-long midcolon pieces were washed with cold PBS and incubated in HBSS (without calcium/magnesium) supplemented with 2% FCS, 10 mM HEPES and 5 mM DTT at 80 r.p.m. and 37 °C for 8 min before being incubated three times in HBSS (without calcium/magnesium) supplemented with 2% FCS, 10 mM HEPES and 5 mM EDTA at 80 r.p.m. at 37 °C for 7 min. Next, the colons were rinsed in HBSS (with calcium/magnesium) supplemented with 2% FCS and 10 mM HEPES at 80 r.p.m. at 37 °C for 5 min. Tissues were then minced using a gentleMACS dissociator (Miltenyi Biotec) in digestion buffer (HBSS (with calcium/magnesium) supplemented with 3% FCS, 10 mM HEPES, 30 μg ml^–1^ DNase I and 100 μg ml^–1^ Liberase and incubated at 120 r.p.m. at 37 °C for 25 min before being filtered through a 100-μm cell strainer and washed with cold PBS.

### Quantitative real-time PCR

RNA from sorted cells was isolated using a Quick-RNA Microprep kit (Zymogen). Reverse transcription of RNA to cDNA was performed using M-MLV reverse transcriptase (Invitrogen). Quantitative real-time PCR was performed on a CFX384 Touch Real-Time PCR Detection System (Bio-Rad) using SYBR Green (Bio-Rad). The following primer pairs were used: *Il23r:* forward CCAAGTATATTGTGCATGTGAAGA, reverse AGCTTGAGGCAAGATATTGTTGT; *Polr2a:* forward CTGGTCCTTCGAATCCGCATC, reverse GCTCGATACCCTGCAGGGTCA.

### Flow cytometry

For intracellular cytokine labeling, cells were restimulated in medium containing ionomycin (500 ng ml^–1^; Invitrogen) and phorbol 12-myristate 13-acetate (50 ng ml^–1^; AppliChem; RPMI complete) with GolgiPlug and GolgiStop (both 1:1,000; BD Biosciences) at 37 °C for 4 h. For surface antibodies, single-cell suspensions were incubated with antibodies in PBS at 4 °C for 20 min. For intranuclear/intracellular stainings, cells were fixed and permeabilized using the eBioscience Foxp3/transcription factor fixation/permeabilization concentrate and diluent, 2% buffered formalin or BD Cytofix at 4 °C for 20 to 35 min. Thereafter, cells were incubated with antibodies in permeabilization buffer (BD) at 4 °C for 30 min, 2 h or overnight.

Viability dyes (1:500 dilution) were either purchased from BioLegend (Zombie NIR) or BD Biosciences (LIVE DEAD Blue). Anti-mouse antibodies, including anti-CD279 (BV785, clone 29F.1A12, 1:200 dilution), anti-ICOS (BV750, clone C398.4A, 1:200 dilution), anti-NK1.1 (BV711, clone PK136, 1:150 dilution), anti-CD25 (BV650, clone PC61, 1:100 dilution), anti-CD152 (BV605, clone UC10-4B9, 1:200 dilution), anti-CD62L (BV570, clone MEL-14, 1:200 dilution), anti-granzyme B (Pacific Blue, clone GB11, 1:50 dilution), anti-neuropilin-1 (BV421, clone 3E+12, 1:200 dilution), anti-CD103 (Biotin, clone 2E7, 1:100 dilution), anti-Helios (PE-Cy7, clone 22F6, dilution 1:30), anti-TCRβ (PE-Cy5, clone H57-597, dilution 1:300), anti-KLRG1 (BV421, clone 2F1/KLRG1, dilution 1:200), anti-KLRG1 (PE-Dazzle 594, clone 2F1/KLRG1, dilution 1:400), anti-CD38 (APC-Fire 810, clone 90, dilution 1:400), anti-CCR8 (Spark NIR 685, clone SA214G2, dilution 1:200), anti-TIM-3 (APC, clone RMT3-23, dilution 1:400), anti-TIM-3 (PE-Fire 810, clone RMT3-23, dilution 1:400), anti-CD4 (Spark NIR 685, clone GK1.5, 1:250 dilution), anti-CD206 (Alexa Fluor 700, clone C068C2, dilution 1:600), anti-F4/80 (APC/Fire750, clone BM8, dilution 1:400), anti-CD86 (PE-Dazzle 594, clone GL1, 1:1,200 dilution), anti-I-A/I-E (PE-Cy5, clone M5/114.15.2, 1:2,000 dilution), anti-CD90.2 (Pacific Blue, clone 30-H12, 1:500 dilution), anti-CD11b (BV510, clone M1/70, 1:1,500 dilution), anti-CD64 (BV605, clone X54-5/7.1, 1:100 dilution), anti-XCR1 (clone ZET, 1:300 dilution), anti-Ly6C (BV711, clone HK1.4, 1:2,000 dilution), anti-CX3CR1 (BV785, clone SA011F11, 1:400 dilution), anti-T-bet (BV711, clone 4B10, 1:50 dilution), anti-IRF4 (Pacific Blue, clone IRF4.3E4, 1:100 dilution), anti-GFP (Alexa Fluor 488, clone FM264G, 1:50 dilution), anti-CD45 (PE-Fire 810, clone S18009F, 1:150 dilution), anti-Ox-40 (APC-Fire750, clone Ox-86, 1:200 dilution), anti-LAG-3 (custom conjugated to NovaFluor Blue 610/70S (dye purchased from Thermo Fisher), clone C9B7W, 1:300 dilution), anti-TNF (BV711, clone MP6-XT22, 1:600 dilution), anti-IL-2 (BV510, clone JES6-5H4, 1:200) and anti-IL-10 (PE-Dazzle 594, clone JES5-16E3, 1:200 dilution), were obtained from BioLegend. Anti-mouse antibodies, including anti-CD69 (BUV395, clone H1.2F3, 1:100 dilution), anti-CD4 (BUV496, clone GK1.5, 1:400 dilution), anti-CD357 (BUV563, clone DTA-1, 1:400 dilution), anti-CD304 (BUV661, clone V46-1954, 1:400 dilution), anti-ST2 (BUV737, clone U29-93, 1:200 dilution), anti-CD8a (BUV805, clone 53-6.7, 1:150 dilution), anti-CD73 (BB660 custom conjugate, clone TY/23, 1:200 dilution), anti-Eomes (PE-CF594, clone X4-83, 1:100 dilution), anti-Eos (PE, clone W7-486, 1:200 dilution), anti-CD27 (R718, clone LG.3A10, 1:200 dilution), anti-Ki-67 (BV480, clone B56, 1:200 dilution), anti-CD44 (BUV737, clone IM7, dilution 1:1,200), anti-Ly6G (BUV563, clone 1A8, 1:700 dilution), anti-CD19 (BUV661, clone 1D3, 1:400 dilution), anti-CD45 (BUV395, clone 30-F11, 1:800 dilution), anti-CD172a (BUV395, clone P84, 1:100 dilution), anti-CD88 (BV750, clone 20/70, 1:200 dilution), anti-NK1.1 (BB700, clone PK136, 1:100 dilution), anti-Siglec-F (BB515, clone E50-2440, 1:2,000 dilution), anti-IL-17A (PE, clone TC11-18H10, 1:600 dilution), anti-pSTAT3 (pY705; PE, clone 4/pSTAT3, 1:200 dilution), anti-pSTAT5 (pY694; Pacific Blue, clone 47/Stat5(pY694), 1:50 dilution), BB630 Streptavidin (custom conjugate, 1:200 dilution) and BUV615 Streptavidin (custom conjugate, 1:200 dilution) were purchased from BD Biosciences. Anti-mouse antibodies, including anti-arginase-1 (APC, clone A1ex5, dilution 1:400), anti-CD11c (PE-Cy5.5, clone N418, 1:1,800 dilution), anti-NOS2 (PE-eFluor610, clone CXNFT, 1:800 dilution), anti-MerTK (PE-Cy7, clone DS5MMER, 1:200 dilution), anti-CD39 (PerCP-eFluor 710, clone 24DMS1, 1:400 dilution), anti-Foxp3 (PE-Cy5.5, clone FJK-16s, 1:200 dilution), anti-IFNγ (PE-Cy7, clone XMG1.2, 1:400 dilution) and anti-IL-22 (APC, clone IL22JOP, 1:200 dilution) were purchased from Thermo Fisher Scientific. Anti-TCF-1 (Alexa Fluor 488, clone C63D9, 1:200 dilution) was obtained from Cell Signaling Technologies. Anti-TOX (PE, clone REA473, 1:200 dilution) was purchased from Miltenyi.

Data were acquired on a 5L Cytek Aurora (Cytek), and data were analyzed using FlowJo software (Tree Star). Cell sorting was performed on a 3L or 5L FACSAria III (BD). Fluorochrome-conjugated monoclonal antibodies were purchased from BioLegend, BD, Thermo Fisher, Miltenyi or Cell Signaling Technologies. For blocking, TruStain FcX (BioLegend; purified anti-CD16/32 (clone 93)) was used. Cellblox Blocking Buffer (Thermo Fisher Scientific) was used to further minimize nonspecific binding.

### In vitro cytokine stimulation of T_reg_ cells

T_reg_ cells derived from *Foxp3*^Cre-YFP^ mice were isolated using a CD4^+^CD25^+^ Regulatory T Cell Isolation kit (Miltenyi) and were cultured in the presence of 2,000 U ml^–1^ recombinant IL-2 (Peprotech) and anti-CD3/CD28 beads of the mouse T_reg_ cell expansion kit (Miltenyi) with a ratio of two beads per cell. In addition, recombinant mouse IL-6 (50 ng ml^–1^; Peprotech), IFNγ (100 ng ml^–1^; Peprotech) and recombinant IL-23 (20 ng ml^–1^; BioLegend) were added, and the cells were cultured for 5 d. For short-term stimulation, T_reg_ cells were, after the 5-d expansion, stimulated with recombinant IL-23 (50 ng ml^–1^; BioLegend) for 30 min. Cells were stained with LIVE/DEAD Zombie NIR for 15 min at 4 °C and fixed using 1:1 fixation concentrate and diluent of the Foxp3 transcription factor kit (Thermo Fisher). The cells were then stained intracellularly with antibodies in perm buffer for 30 min at room temperature in the dark.

### In vitro cultivation of human T_reg_ cells

T_reg_ cells were sorted by FACS from freshly isolated human peripheral blood mononuclear cells (gated on FOXP3^+^CD45^+^CD3^+^CD4^+^CD27^+^CD25^+^CD127^–^) and incubated in T_reg_ cell culture medium containing DMEM supplemented with 10% fetal bovine serum, 1× penicillin/streptomycin (Gibco), 1× MEM vitamin solution (Gibco), 1 mM sodium pyruvate (Gibco), 1× MEM non-essential amino acid solution (Gibco), 100 mM HEPES (Gibco), 0.5 mM 2-mercaptoethanol (Gibco), 1× GlutaMAX (Gibco), recombinant human IL-2 500 U ml^–1^ (Peprotech) and anti-CD3/CD28 stimulation beads (four beads per cell; human T_reg_ cell expansion kit; Miltenyi) at 37 °C for 2 d. CD45^+^CD3^+^CD4^+^CD25^+^CD27^+^FOXP3^+^ cells were defined as T_reg_ cells (human) by flow cytometry.

### Seahorse assay

Mouse T_reg_ cells of *Foxp3*^Cre-YFP^*Il23r*^fl/fl^ or *Foxp3*^Cre-YFP^ mice were isolated from steady-state spleens and LNs by using the mouse T_reg_ cell isolation kit according to the manufacturer’s protocol (Miltenyi). Purity was above 90% (assessed by flow cytometry). Isolated T_reg_ cells were cultured in T_reg_ cell culture medium and stimulated using the mouse T_reg_ cell expansion kit (Miltenyi) with four beads per cell and 2,000 U ml^–1^ recombinant mouse IL-2 (Peprotech) for 3 d. The ECAR of cultured T_reg_ cells was measured in a 96-well XFe Extracellular Flux Analyzer (Agilent)^62^. One hundred and fifty thousand T_reg_ cells were starved and plated per well in XF medium (non-buffered RPMI-1640 (Agilent) supplemented with 2 mM l-glutamine) at 37 °C for 30 min. The respective wells were treated with recombinant mouse IL-23 (50 ng ml^–1^; Peprotech) in the Seahorse plate 20 min before measurements. ECAR was investigated at the basal level after glucose addition (final concentration of 10 mM) in response to oligomycin (final concentration of 1 μM) and after 2-deoxyglucose (final concentration of 50 mM). Glycolysis was calculated as maximum rate measurement before oligomycin injection – last rate measurement before glucose injection.

### In vitro T_reg_ cell suppression assay

For in vitro T_reg_ suppression assays^63^, red blood cell lysis was performed using RBC lysis buffer (Abcam) on splenocytes of *Foxp3*^Cre-YFP^ mice at room temperature for 2 min. CD4^+^ T cells were enriched using a CD4^+^ T cell isolation kit (Miltenyi), and T_reg_ cells were then purified by FACS. T_reg_ cells were cultured and preactivated in vitro in the presence of 2,000 U ml^–1^ recombinant IL-2 (Miltenyi) and CD3/CD28 Dynabeads for 3 d (ref. ^64^) to induce eT_reg_ cell differentiation.

Antigen-presenting cells were enriched by depleting CD90.2^+^ splenocytes, exposed to 20 µg ml^–1^ mitomycin C (Sigma) for 30 min at 37 °C and washed five times with PBS. Antigen-presenting cells were plated at 2 × 10^5^ cells per well and used for co-stimulation, and 1 µg ml^–1^ anti-CD3 (clone 17A2) was added for polyclonal TCR activation. CD4^+^ T cells were isolated using a naive T cell isolation kit (Miltenyi) and labeled with CellTrace Violet according to the manufacturer’s protocol, and 2.5 × 10^4^ cells were seeded per well in 96-well plates. T_reg_ cells were added at the indicated ratios, and the assay was performed for 72 h. For antibody treatment, 10 µg ml^–1^ anti-mouse IL-23R (clone 12B2B64) was added. Percent suppression was assessed based on the division index (DI) calculated in FlowJo with the following formula: percent suppression = 100 – (DI_Treg:Tcon ratio_/DI_Tcon alone_) × 100 (ref. ^65^).

### In vivo cytokine blockade

Six- to 8-week-old C57BL/6 mice were subcutaneously inoculated with 3 × 10^5^ MC38 cells. The mice were randomized to respective treatment groups on day 6 after inoculation and received a total of three injections of 100 µg of anti-p19 (clone G23-8, BioXcell) or isotype control (IgG1, clone MOPC-21, BioXcell) every 72 h intraperitoneally.

### Histology

For immunofluorescence stainings, tissues were fixed in 4% paraformaldehyde at 4 °C for 24 h. Tissues were then put into PBS with 30% sucrose at 4 °C for 72 h and embedded in optimal cutting temperature compound. Cut sections were incubated with working solution (PBS supplemented with 1% bovine serum albumin and 0.02% Tween 20) at 4 °C for 30 min. Sections were then incubated with primary antibodies to Foxp3 and tdTomato diluted in working solution at 4 °C overnight. Sections were washed three times with PBS supplemented with 0.01% Tween for 5 min and were incubated with secondary antibodies and DAPI diluted in working solution at 4 °C for 30 min, followed by another round of five wash steps with PBS and 0.01% Tween 20. Image acquisition was performed on a Leica Stellaris 5.

### High-dimensional analysis of flow cytometry data

Raw fcs-files were preprocessed using FlowJo Software. Compensated and pregated cells were imported into RStudio using R (version 4.0/4.2.2) and the flowCore package^66^. Data were transformed using a hyperbolic arcsine (arcsinh) transformation and percentile normalized to obtain expression values between 0 and 1. This was followed by dimensionality reduction using UMAP by applying the umap package in R^25^. Automated clustering and metaclustering were performed with the FlowSOM algorithm^26^. This was followed by expert-guided merging of clusters^67^.

### scRNA-seq

Mouse tumors were digested as described above. CD4^+^ and CD8^+^ T cell enrichment was then performed using CD4/CD8 (TIL) MicroBeads (Milteny Biotec) following the manufacturer’s instructions. Enriched cells were labeled with flow cytometry antibodies in PBS at 4 °C for 20 min. After a wash step with PBS supplemented with 2% FCS, cells were labeled with antibody-seq oligonucleotides (BD) and sample tag antibodies to MHC class I (626545 BD Single-Cell Multiplexing Kit) at 4 °C for 45 min in PBS supplemented with 2% FCS. Antibody-seq oligonucleotides, including anti-CD27 (clone LG.3A10), anti-CD4 (clone GK1.5), anti-CD103 (clone 2E7), anti-CD357 (clone DTA-1), anti-CD8a (clone 53-6.7), anti-CD279 (clone RMP1-30), anti-CD44 (clone IM7), anti-CD25 (clone PC6), anti-CD62L (clone MEL-14), anti-CD45RA (clone 14.8), anti-KLRG1 (clone 2F1), anti-ICOS (clone DX-29) and anti-CD38 (clone 90/CD38), were obtained from BD Biosciences.

Cells were then washed three times with PBS supplemented with 2% FCS, and CD45^+^CD90^+^CD4^+^ live cells were sorted by FACS into RPMI supplemented with 5% FCS. Sorted CD4^+^ T cells were washed once with PBS supplemented with 2% FCS, and six sample tag-labeled samples were multiplexed to obtain a total of 60,000 cells, which were loaded on a BD Rhapsody cartridge. Single-cell isolation was performed with the BD Rhapsody Express Single-Cell Analysis system according to the manufacturer’s protocol (BD Biosciences). Targeted cDNA library preparation was conducted with the targeted mRNA and AbSeq amplification kit (BD Biosciences), the BD Rhapsody Immune Response Panel and a complementary custom-designed targeted panel. Size distribution of the cDNA libraries was performed using a D1000 assay on a TapeStation system (Agilent Technologies). Sequencing was performed on a Novaseq S1 (Illumina) by the Functional Genomics Center Zurich.

### scRNA-seq analysis

Raw sequencing reads were uploaded to the SevenBridges analysis platform. For each sample, the BD Rhapsody targeted analysis pipeline (revision 0) was run using a custom amplicon and AbSeq antibody-tag reference. All other app defaults were left unchanged.

Downstream analysis was performed using the Seurat (4.1.0/4.2.0), SingleCellExperiment (version 1.20.0) and scater (version 1.26.1) packages. Cells with <200 or >2,500 genes were excluded from further analysis.

The data were log normalized and scaled and underwent principal component analysis (PCA) based on all features. Subsequently, clustering and UMAP dimensionality reduction was performed based on 30 principal components and a resolution of 1.2. The clusters were then manually assigned based on their differential marker expression. The identified T_reg_ cell cluster was subsetted, and log normalization, scaling and PCA were performed on the subsetted data as described above. For differential expression analysis, a non-parametric Wilcoxon rank-sum test was performed. Tumor and tdLN data were integrated via the Seurat v4 pipeline using SelectIntegrationFeatures, FindIntegrationAnchors and IntegrateData, followed by identical processing as described above. For trajectory inference analysis, the integrated Seurat object was converted into a Monocle 3 cell_data_set (cds) object including previous embedding and clustering information. Using learn_graph and order_cells, a principal graph was fitted on the data, and the cells were ordered along a pseudotemporal trajectory with an automatic selection of the root node^68–70^.

The AddModuleScore function was used to compute cluster-specific scores. The SCpubr package^71^ (version 1.0.4) was used for visualizations of subset markers and cellular state plots. For the cellular state plots, enrichment scores for each cluster were computed using the AddModuleScore function implemented in Seurat based on the 30 most DEGs between the identified T_reg_ cell clusters. The do_CellularStatesPlot function of the SCpubr package was then leveraged to visualize the enrichment scores. DA testing based on partially overlapping graph neighborhoods was performed using the Milo package (version 1.7.0)^38^. Subsetted T_reg_ cells were grouped in *Il23r*-KO and WT T_reg_ cells based on their expression of *Yfp-cre*, and the Seurat object was converted into a SingleCellExperiment object before the Milo object was generated. A *k*-nearest neighbors graph with 12 reduced dimensions and *k* = 10 for *k*-nearest neighbors refinement was applied. To perform DA testing, a design matrix with *YFP* positivity as a covariate to test for was applied. The built-in visualization functions of Milo were then used to generate DA plots.

Publicly available scRNA-seq datasets were analyzed using the Seurat (4.1.0/4.2.0) package in RStudio. Briefly, if available, clustering of publicly available data was used, and expert-guided merging of clusters was performed in some cases. Otherwise, expert-guided manual cell-type assignment to the unbiased clusters was performed. Differential expression was assessed using the non-parametric Wilcoxon rank-sum test.

Cell–cell communication network inference was performed on myeloid and T_reg_ cell subsets extracted with Seurat using the ICELLNET (version 1.00) packages in RStudio^42,72^.

### Bulk RNA-seq analysis

Differential expression analysis on publicly available bulk RNA-seq datasets was performed using the DESeq2 package (version 1.37.4)^73^. Briefly, unnormalized count matrices were imported into RStudio using R version 4.0. Prefiltering of low-count genes was performed by selecting only genes with ten or more reads. A DESeqDataSet object was then generated using the DESeqDataSetFromMatrix() function with design ~ condition (for example, tumor T_reg_ cells versus spleen T_reg_ cells). Rows with low gene counts (less than five) were removed in the next step. The DESeq function was then applied with default parameters, and the results were filtered for an adjusted *P* value of <0.05 and log_2_ (fold change) of >1.5. Consequently, a *z* score was calculated on these genes.

### Quantification and statistical analysis

Statistical significance was determined using GraphPad Prism 8 (GraphPad Software). Two-tailed, unpaired *t*-tests were used to assess differences between two groups. Statistical significance for disease curves was evaluated by two-way ANOVA with Bonferroni’s post hoc test. *n* shows the number of biological replicates. Statistical details for each experiment are indicated in the corresponding figure legends.

### Reporting summary

Further information on research design is available in the Nature Portfolio Reporting Summary linked to this article.

## Online content

Any methods, additional references, Nature Portfolio reporting summaries, source data, extended data, supplementary information, acknowledgements, peer review information; details of author contributions and competing interests; and statements of data and code availability are available at 10.1038/s41590-024-01755-7.

### Supplementary information

Reporting Summary

Supplementary Table 1 *Foxp3*^Cre-YFP/+^ (heterozygous) *Il23r*^fl/fl^ female mice were inoculated i.d. with B16 tumor cells, and combined transcriptome (scRNA-seq) and protein expression analysis of sorted CD4^+^ T cells was performed on day 13 after inoculation. A list of subset markers for each T_reg_ cell subset was generated with the FindAllMarkers function in Seurat. A two-sided Wilcoxon rank-sum test with a Bonferroni correction was used for multiple testing.

Supplementary Table 2 List of DEGs between T_reg_ cell subsets (adjusted *P* value of <0.05). A two-sided Wilcoxon rank-sum test with a Bonferroni correction was used for multiple testing.

Supplementary Table 3 Analyses of a human pan-cancer single-cell sequencing dataset from Zheng et al.^41^. List of DEGs between *IL23R*^high^ (*IL23R* expression > 0) and *IL23R*^low^ (*IL23R* expression = 0) T_reg_ cells (adjusted *P* value of < 0.05). A two-sided Wilcoxon rank-sum test with a Bonferroni correction was used for multiple testing.

Supplementary Table 4 Analyses of a human pan-cancer single-cell sequencing dataset from Zheng et al.^41^. KEGG pathways were identified by comparing DEGs between *IL23R*^high^ (*IL23R* expression > 0) and *IL23R*^low^ (*IL23R* expression = 0) T_reg_ cells. A two-sided Wilcoxon rank-sum test with a Bonferroni correction was used for multiple testing.

Supplementary Table 5 Top genes per cluster for scRNA-seq datasets analyzed in the study.

### Source data

Source Data Fig. 1 Statistical source data.

Source Data Fig. 2 Statistical source data.

Source Data Fig. 3 Statistical source data.

Source Data Fig. 4 Statistical source data.

Source Data Fig. 6 Statistical source data.

Source Data Extended Data Fig. 1 Statistical source data.

Source Data Extended Data Fig. 2 Statistical source data.

Source Data Extended Data Fig. 3 Statistical source data.

Source Data Extended Data Fig. 4 Statistical source data.

## Data Availability

scRNA-seq data generated for this study have been deposited in the Gene Expression Omnibus (https://www.ncbi.nlm.nih.gov/geo/) under accession number GSE224072. Human TIL data from a pan-cancer T cell atlas are available under the accession number GSE156728. scRNA-seq data from tumor-infiltrating leukocytes from individuals with colorectal cancer are accessible under the accession number GSE164522. Bulk RNA-seq data of mouse and human tumor-infiltrating T_reg_ cells are accessible under the accession number GSE116347. Source data are provided with this paper.

## Code availability

Code will be provided upon reasonable request by the corresponding authors.

## References

[1] Sakaguchi S (2020). Regulatory T cells and human disease. Annu. Rev. Immunol..

[2] Rosenblum MD, Way SS, Abbas AK (2016). Regulatory T cell memory. Nat. Rev. Immunol..

[3] Tanaka A, Sakaguchi S (2017). Regulatory T cells in cancer immunotherapy. Cell Res..

[4] Teh PP, Vasanthakumar A, Kallies A (2015). Development and function of effector regulatory T cells. Prog. Mol. Biol. Transl. Sci..

[5] Labani-Motlagh A, Ashja-Mahdavi M, Loskog A (2020). The tumor microenvironment: a milieu hindering and obstructing antitumor immune responses. Front. Immunol..

[6] Iorgulescu JB, Braun D, Oliveira G, Keskin DB, Wu CJ (2018). Acquired mechanisms of immune escape in cancer following immunotherapy. Genome Med..

[7] Arce Vargas F (2017). Fc-optimized anti-CD25 depletes tumor-infiltrating regulatory T cells and synergizes with PD-1 blockade to eradicate established tumors. Immunity.

[8] Klages, K. et al. Selective depletion of Foxp3^+^ regulatory T cells improves effective therapeutic vaccination against established melanoma. *Cancer Res*. **70**, 7788–7799 (2010). 10.1158/0008-5472.CAN-10-1736 20924102

[9] Onizuka S (1999). Tumor rejection by in vivo administration of anti-CD25 (interleukin-2 receptor α) monoclonal antibody. Cancer Res..

[10] Ohue Y, Nishikawa H (2019). Regulatory T (T_reg_) cells in cancer: can T_reg_ cells be a new therapeutic target?. Cancer Sci..

[11] Tang C, Chen S, Qian H, Huang W (2012). Interleukin-23: as a drug target for autoimmune inflammatory diseases. Immunology.

[12] Croxford AL, Mair F, Becher B (2012). IL-23: one cytokine in control of autoimmunity. Eur. J. Immunol..

[13] Zwicky P, Unger S, Becher B (2020). Targeting interleukin-17 in chronic inflammatory disease: a clinical perspective. J. Exp. Med..

[14] Kortylewski M (2009). Regulation of the IL-23 and IL-12 balance by Stat3 signaling in the tumor microenvironment. Cancer Cell.

[15] Langowski JL (2006). IL-23 promotes tumour incidence and growth. Nature.

[16] Teng MWL (2010). IL-23 suppresses innate immune response independently of IL-17A during carcinogenesis and metastasis. Proc. Natl Acad. Sci. USA.

[17] Teng MWL, Von Scheidt B, Duret H, Towne JE, Smyth MJ (2011). Anti-IL-23 monoclonal antibody synergizes in combination with targeted therapies or IL-2 to suppress tumor growth and metastases. Cancer Res..

[18] Mujal AM (2022). Holistic characterization of tumor monocyte-to-macrophage differentiation integrates distinct immune phenotypes in kidney cancer. Cancer Immunol. Res..

[19] Andreatta M (2021). Interpretation of T cell states from single-cell transcriptomics data using reference atlases. Nat. Commun..

[20] Awasthi, A. et al. IL-23 receptor GFP reporter mice reveal distinct populations of IL-17-producing cells. *J. Immunol.* **182**, 5904–5908 (2009). 10.4049/jimmunol.0900732 PMC2702203 19414740

[21] Yoon J (2016). IL-23 induced in keratinocytes by endogenous TLR4 ligands polarizes dendritic cells to drive IL-22 responses to skin immunization. J. Exp. Med..

[22] Wight AE (2022). Antibody-mediated blockade of the IL23 receptor destabilizes intratumoral regulatory T cells and enhances immunotherapy. Proc. Natl Acad. Sci. USA.

[23] Jones LL, Alli R, Li B, Geiger TL (2016). Differential T cell cytokine receptivity and not signal quality distinguishes IL-6 and IL-10 signaling during T_H_17 differentiation. J. Immunol..

[24] Malik S, Want MY, Awasthi A (2016). The emerging roles of γδ T cells in tissue inflammation in experimental autoimmune encephalomyelitis. Front. Immunol..

[25] McInnes L, Healy J, Melville J (2018). UMAP: uniform manifold approximation and projection for dimension reduction. J. Open Source Softw..

[26] Van Gassen, S. et al. FlowSOM: using self-organizing maps for visualization and interpretation of cytometry data. *Cytometry A* **87**, 636–645 (2015). 10.1002/cyto.a.22625 25573116

[27] Magnuson AM (2018). Identification and validation of a tumor-infiltrating T_reg_ transcriptional signature conserved across species and tumor types. Proc. Natl Acad. Sci. USA.

[28] Philip, M. & Schietinger, A. CD8^+^ T cell differentiation and dysfunction in cancer. *Nat. Rev. Immunol.* **22**, 209–223 (2022). 10.1038/s41577-021-00574-3 PMC9792152 34253904

[29] Debacker JM, Gondry O, Lahoutte T, Keyaerts M, Huvenne W (2021). The prognostic value of CD206 in solid malignancies: a systematic review and meta-analysis. Cancers.

[30] Ekmekcioglu S, Grimm EA, Roszik J (2017). Targeting iNOS to increase efficacy of immunotherapies. Hum. Vaccin. Immunother..

[31] Niu F (2022). Arginase: an emerging and promising therapeutic target for cancer treatment. Biomed. Pharmacother..

[32] Fontenot JD (2005). Regulatory T cell lineage specification by the forkhead transcription factor Foxp3. Immunity.

[33] Garg G (2019). Blimp1 prevents methylation of Foxp3 and loss of regulatory T cell identity at sites of inflammation. Cell Rep..

[34] Zappasodi R (2021). CTLA-4 blockade drives loss of T_reg_ stability in glycolysis-low tumours. Nature.

[35] Gerriets VA (2016). Foxp3 and Toll-like receptor signaling balance T_reg_ cell anabolic metabolism for suppression. Nat. Immunol..

[36] Wei J (2016). Autophagy enforces functional integrity of regulatory T cells by coupling environmental cues and metabolic homeostasis. Nat. Immunol..

[37] Dixon ML, Leavenworth JD, Leavenworth JW (2021). Lineage reprogramming of effector regulatory T cells in cancer. Front. Immunol..

[38] Dann E, Henderson NC, Teichmann SA, Morgan MD, Marioni JC (2021). Differential abundance testing on single-cell data using *k*-nearest neighbor graphs. Nat. Biotechnol..

[39] Munn DH, Sharma MD, Johnson TS (2018). T_reg_ destabilization and reprogramming: implications for cancer immunotherapy. Cancer Res..

[40] Cheng S (2021). A pan-cancer single-cell transcriptional atlas of tumor infiltrating myeloid cells. Cell.

[41] Zheng L (2021). Pan-cancer single-cell landscape of tumor-infiltrating T cells. Science.

[42] Noël F (2021). Dissection of intercellular communication using the transcriptome-based framework ICELLNET. Nat. Commun..

[43] Liu Y (2022). Immune phenotypic linkage between colorectal cancer and liver metastasis. Cancer Cell.

[44] Revel M, Sautès-Fridman C, Fridman WH, Roumenina LT (2022). C1q^+^ macrophages: passengers or drivers of cancer progression. Trends Cancer.

[45] Hu JM (2017). CD163 as a marker of M2 macrophage, contribute to predict aggressiveness and prognosis of Kazakh esophageal squamous cell carcinoma. Oncotarget.

[46] Kanehisa M, Sato Y, Kawashima M, Furumichi M, Tanabe M (2016). KEGG as a reference resource for gene and protein annotation. Nucleic Acids Res..

[47] Reimand J (2016). g:Profiler—a web server for functional interpretation of gene lists (2016 update). Nucleic Acids Res..

[48] Doebbeler M (2018). CD83 expression is essential for T_reg_ cell differentiation and stability. JCI Insight.

[49] Mijnheer G (2021). Conserved human effector T_reg_ cell transcriptomic and epigenetic signature in arthritic joint inflammation. Nat. Commun..

[50] Alvisi G (2020). IRF4 instructs effector T_reg_ differentiation and immune suppression in human cancer. J. Clin. Investig..

[51] Qureshi OS (2011). *Trans*-endocytosis of CD80 and CD86: a molecular basis for the cell-extrinsic function of CTLA-4. Science.

[52] Tekguc M, Wing JB, Osaki M, Long J, Sakaguchi S (2021). T_reg_-expressed CTLA-4 depletes CD80/CD86 by trogocytosis, releasing free PD-L1 on antigen-presenting cells. Proc. Natl Acad. Sci. USA.

[53] Akkaya B (2019). Regulatory T cells mediate specific suppression by depleting peptide–MHC class II from dendritic cells. Nat. Immunol..

[54] Tiemessen MM (2007). CD4^+^CD25^+^Foxp3^+^ regulatory T cells induce alternative activation of human monocytes/macrophages. Proc. Natl Acad. Sci. USA.

[55] Jain R (2016). Interleukin-23-induced transcription factor Blimp-1 promotes pathogenicity of T helper 17 cells. Immunity.

[56] Dixon ML (2021). Remodeling of the tumor microenvironment via disrupting Blimp1^+^ effector T_reg_ activity augments response to anti-PD-1 blockade. Mol. Cancer.

[57] Ono M (2007). Foxp3 controls regulatory T-cell function by interacting with AML1/Runx1. Nature.

[58] Di Pilato M (2019). Targeting the CBM complex causes T_reg_ cells to prime tumours for immune checkpoint therapy. Nature.

[59] Mair F (2022). Extricating human tumour immune alterations from tissue inflammation. Nature.

[60] Aden K (2016). Epithelial IL-23R signaling licenses protective IL-22 responses in intestinal inflammation. Cell Rep..

[61] Zwicky P (2021). IL-12 regulates type 3 immunity through interfollicular keratinocytes in psoriasiform inflammation. Sci. Immunol..

[62] Uhl FM (2020). Metabolic reprogramming of donor T cells enhances graft-versus-leukemia effects in mice and humans. Sci. Transl. Med..

[63] Overacre-Delgoffe AE (2017). Interferon-γ drives T_reg_ fragility to promote anti-tumor immunity. Cell.

[64] Collison, L. W. & Vignali, D. A. A. in *Regulatory T Cells*, Vol. 707 (eds Kassiotis, G. & Liston, A.) 21–37 (Humana Press, 2011).

[65] McMurchy AN, Levings MK (2012). Suppression assays with human T regulatory cells: a technical guide. Eur. J. Immunol..

[66] Ellis, B., et al. flowCore: flowCore: basic structures for flow cytometry data. R package version 2.12.2. 10.18129/B9.bioc.flowCore (2023).

[67] Ingelfinger, F. et al. Single-cell profiling of myasthenia gravis identifies a pathogenic T cell signature. *Acta Neuropathol.* **141**, 901–915 (2021). 10.1007/s00401-021-02299-y PMC8113175 33774709

[68] Trapnell C (2014). The dynamics and regulators of cell fate decisions are revealed by pseudotemporal ordering of single cells. Nat. Biotechnol..

[69] Qiu X (2017). Reversed graph embedding resolves complex single-cell trajectories. Nat. Methods.

[70] Cao J (2019). The single-cell transcriptional landscape of mammalian organogenesis. Nature.

[71] Blanco-Carmona, E. Generating publication ready visualizations for single cell transcriptomics using SCpubr. Preprint at *bioRxiv*10.1101/2022.02.28.482303 (2022).

[72] Jin S (2021). Inference and analysis of cell–cell communication using CellChat. Nat. Commun..

[73] Love MI, Huber W, Anders S (2014). Moderated estimation of fold change and dispersion for RNA-seq data with DESeq2. Genome Biol..

