# Supplementary material for: IL-23 stabilizes an effector Treg cell program in the tumor microenvironment
Source: Nat Immunol. 2024 Feb 14;25(3):512–24. doi: 10.1038/s41590-024-01755-7 (PMC10907296; doi:10.1038/s41590-024-01755-7)
Supplement: Supplementary file 1 — Reporting Summary [file 41590_2024_1755_MOESM1_ESM.pdf]

Corresponding author(s): Burkhard Becher
Sonia Tugues

Last updated by author(s): Jan 20, 2023

# Reporting Summary

Nature Portfolio wishes to improve the reproducibility of the work that we publish. This form provides structure for consistency and transparency in reporting. For further information on Nature Portfolio policies, see our Editorial Policies and the Editorial Policy Checklist.

## Statistics

For all statistical analyses, confirm that the following items are present in the figure legend, table legend, main text, or Methods section.

| n/a | Confirmed | |
|---|---|---|
| ☐ | ☒ | The exact sample size (*n*) for each experimental group/condition, given as a discrete number and unit of measurement |
| ☐ | ☒ | A statement on whether measurements were taken from distinct samples or whether the same sample was measured repeatedly |
| ☐ | ☒ | The statistical test(s) used AND whether they are one- or two-sided<br>*Only common tests should be described solely by name; describe more complex techniques in the Methods section.* |
| ☐ | ☒ | A description of all covariates tested |
| ☐ | ☒ | A description of any assumptions or corrections, such as tests of normality and adjustment for multiple comparisons |
| ☐ | ☒ | A full description of the statistical parameters including central tendency (e.g. means) or other basic estimates (e.g. regression coefficient) AND variation (e.g. standard deviation) or associated estimates of uncertainty (e.g. confidence intervals) |
| ☐ | ☒ | For null hypothesis testing, the test statistic (e.g. *F*, *t*, *r*) with confidence intervals, effect sizes, degrees of freedom and *P* value noted<br>*Give P values as exact values whenever suitable.* |
| ☒ | ☐ | For Bayesian analysis, information on the choice of priors and Markov chain Monte Carlo settings |
| ☒ | ☐ | For hierarchical and complex designs, identification of the appropriate level for tests and full reporting of outcomes |
| ☐ | ☒ | Estimates of effect sizes (e.g. Cohen's *d*, Pearson's *r*), indicating how they were calculated |

*Our web collection on statistics for biologists contains articles on many of the points above.*

## Software and code

Policy information about availability of computer code

| | |
|---|---|
| Data collection | Flow cytometry data: Cytek Aurora 5L; SpectroFlo Software (v3.0 & v3.1).<br>BD FACSAria™ 3L or 5L , FACS Diva Software v9.1.<br>Immunofluorescence: Leica SP5 confocal microscope.<br>BD Rhapsody single cell RNA sequencing: mouse cells were sorted using BD FACSAria™ 5L and loaded on the BD Rhapsody cartridges. Libraries were prepared according to the manufacturer's instructions and sequenced on an Illumina Novaseq S1. |
| Data analysis | Flow cytometry data:<br>FlowJo software (v10, BD)<br>Prism software (GraphPad v9)<br>R-studio (v4.0 & v4.2.2). Mapping and clustering were performed using FlowSOM.<br><br>Single Cell RNA Sequencing:<br>R (v4.0; v4.2.2), Seurat (v4.1.0 and v4.2.0) and scater (v. 1.26.1) for single cell RNA sequencing analyses. Milo (v1.7.0) for neighborhood analysis of mouse Tregs. SCpubr (v.1.0.4) for visualization of scRNAseq data. ICELLNET (v.1.00) for cell-cell communication analysis between myeloid cells and Tregs in human colorectal cancer. DESeq2 (v.1.37.4) for analysis of bulk RNA sequencing data. Monocle 3 was used for trajectory analysis. |

For manuscripts utilizing custom algorithms or software that are central to the research but not yet described in published literature, software must be made available to editors and reviewers. We strongly encourage code deposition in a community repository (e.g. GitHub). See the Nature Portfolio guidelines for submitting code & software for further information.

# Data

Policy information about availability of data

All manuscripts must include a data availability statement. This statement should provide the following information, where applicable:

- Accession codes, unique identifiers, or web links for publicly available datasets
- A description of any restrictions on data availability
- For clinical datasets or third party data, please ensure that the statement adheres to our policy

Single-cell sequencing data generated for this study have been deposited in the Gene Expression Omnibus (GEO) under accession number GSE224072. The accessibility of publicly available datasets used in this study is as follows : Human tumor-infiltrating T cell data from a pan cancer T cell atlas is available under the accession number GSE156728, scRNA-seq data from tumor-infiltrating leucocytes of colorectal cancer patients is accessible under the accession number GSE164522, bulk-RNA sequencing data of mouse and human tumor-infiltrating Tregs is accessible under the accesion number GSE116347.
Other data will be available from the corresponding authors upon request.

# Human research participants

Policy information about studies involving human research participants and Sex and Gender in Research.

| | |
|---|---|
| Reporting on sex and gender | NA |
| Population characteristics | NA |
| Recruitment | NA |
| Ethics oversight | NA |

Note that full information on the approval of the study protocol must also be provided in the manuscript.

# Field-specific reporting

Please select the one below that is the best fit for your research. If you are not sure, read the appropriate sections before making your selection.

☒ Life sciences ☐ Behavioural & social sciences ☐ Ecological, evolutionary & environmental sciences

For a reference copy of the document with all sections, see nature.com/documents/nr-reporting-summary-flat.pdf

# Life sciences study design

All studies must disclose on these points even when the disclosure is negative.

| | |
|---|---|
| Sample size | We did not perform sample size calculations. The sample size in flow cytometry experiments was greater than or equal to 3 mice per group. This is widely accepted in the field of immunology, if 2-3 independent experiments are performed. For single cell RNA sequencing processed on the BD Rhapsody platform 6 biological replicates were chosen, since only one independent experiment was performed. The chosen sample sizes are commonly used in the field of immunology and are similar to those in previous publications (Kim et al. Nature Immunology 2023, Wang et al. Nature Immunology 2020). |
| Data exclusions | No animals were excluded from the analysis. In the B16 experiment presented in figure 3 and MC38 experiment presented in Extended Data Figure 3 we excluded one outlier sample in each experiment, which we identified leveraging the ROUT method for detection of significant statistical outliers.<br>For single cell RNA sequencing, Seven Bridges analysis was used to identify multiplets and cells that could not be linked to a sample tag. These cells were then excluded from further analysis. |
| Replication | Experiments were succesfully repeated and the number of experiments is stated in the figure legends. Cytokine staining in MC38 model was performed once, as we used at least 7 mice per group and observed the same tumor phenotype as in the other tumor models. This also complies with the 3R principle in animal research.<br><br>scRNA sequencing was performed once, since sequencing results are highly robust due to the high number of individual cells, which are analyzed. In addition, scRNAseq experiments are cost-intensive and therefore it is common practice to only perform them once. |
| Randomization | Mice were grouped by genotype (when using Foxp3-Cre/Il23r floxed and Il23r floxed or Foxp3-Cre control mice), age and sex. |
| Blinding | Tumor inoculations and measurement of tumor size by caliper gauge were performed in a blinded fashion. |

# Behavioural & social sciences study design

All studies must disclose on these points even when the disclosure is negative.

| | |
|---|---|
| Study description | *Briefly describe the study type including whether data are quantitative, qualitative, or mixed-methods (e.g. qualitative cross-sectional, quantitative experimental, mixed-methods case study).* |
| Research sample | *State the research sample (e.g. Harvard university undergraduates, villagers in rural India) and provide relevant demographic information (e.g. age, sex) and indicate whether the sample is representative. Provide a rationale for the study sample chosen. For studies involving existing datasets, please describe the dataset and source.* |
| Sampling strategy | *Describe the sampling procedure (e.g. random, snowball, stratified, convenience). Describe the statistical methods that were used to predetermine sample size OR if no sample-size calculation was performed, describe how sample sizes were chosen and provide a rationale for why these sample sizes are sufficient. For qualitative data, please indicate whether data saturation was considered, and what criteria were used to decide that no further sampling was needed.* |
| Data collection | *Provide details about the data collection procedure, including the instruments or devices used to record the data (e.g. pen and paper, computer, eye tracker, video or audio equipment) whether anyone was present besides the participant(s) and the researcher, and whether the researcher was blind to experimental condition and/or the study hypothesis during data collection.* |
| Timing | *Indicate the start and stop dates of data collection. If there is a gap between collection periods, state the dates for each sample cohort.* |
| Data exclusions | *If no data were excluded from the analyses, state so OR if data were excluded, provide the exact number of exclusions and the rationale behind them, indicating whether exclusion criteria were pre-established.* |
| Non-participation | *State how many participants dropped out/declined participation and the reason(s) given OR provide response rate OR state that no participants dropped out/declined participation.* |
| Randomization | *If participants were not allocated into experimental groups, state so OR describe how participants were allocated to groups, and if allocation was not random, describe how covariates were controlled.* |

# Ecological, evolutionary & environmental sciences study design

All studies must disclose on these points even when the disclosure is negative.

| | |
|---|---|
| Study description | *Briefly describe the study. For quantitative data include treatment factors and interactions, design structure (e.g. factorial, nested, hierarchical), nature and number of experimental units and replicates.* |
| Research sample | *Describe the research sample (e.g. a group of tagged Passer domesticus, all Stenocereus thurberi within Organ Pipe Cactus National Monument), and provide a rationale for the sample choice. When relevant, describe the organism taxa, source, sex, age range and any manipulations. State what population the sample is meant to represent when applicable. For studies involving existing datasets, describe the data and its source.* |
| Sampling strategy | *Note the sampling procedure. Describe the statistical methods that were used to predetermine sample size OR if no sample-size calculation was performed, describe how sample sizes were chosen and provide a rationale for why these sample sizes are sufficient.* |
| Data collection | *Describe the data collection procedure, including who recorded the data and how.* |
| Timing and spatial scale | *Indicate the start and stop dates of data collection, noting the frequency and periodicity of sampling and providing a rationale for these choices. If there is a gap between collection periods, state the dates for each sample cohort. Specify the spatial scale from which the data are taken* |
| Data exclusions | *If no data were excluded from the analyses, state so OR if data were excluded, describe the exclusions and the rationale behind them, indicating whether exclusion criteria were pre-established.* |
| Reproducibility | *Describe the measures taken to verify the reproducibility of experimental findings. For each experiment, note whether any attempts to repeat the experiment failed OR state that all attempts to repeat the experiment were successful.* |
| Randomization | *Describe how samples/organisms/participants were allocated into groups. If allocation was not random, describe how covariates were controlled. If this is not relevant to your study, explain why.* |
| Blinding | *Describe the extent of blinding used during data acquisition and analysis. If blinding was not possible, describe why OR explain why blinding was not relevant to your study.* |

Did the study involve field work? ☐ Yes ☒ No

# Reporting for specific materials, systems and methods

We require information from authors about some types of materials, experimental systems and methods used in many studies. Here, indicate whether each material, system or method listed is relevant to your study. If you are not sure if a list item applies to your research, read the appropriate section before selecting a response.

## Materials & experimental systems

| n/a | Involved in the study |
|---|---|
| ☐ | ☒ Antibodies |
| ☐ | ☒ Eukaryotic cell lines |
| ☒ | ☐ Palaeontology and archaeology |
| ☐ | ☒ Animals and other organisms |
| ☒ | ☐ Clinical data |
| ☒ | ☐ Dual use research of concern |

## Methods

| n/a | Involved in the study |
|---|---|
| ☒ | ☐ ChIP-seq |
| ☐ | ☒ Flow cytometry |
| ☒ | ☐ MRI-based neuroimaging |

## Antibodies

Antibodies used

Anti-mouse antibodies including anti-CD279 (BV785, clone 29F.1A12,1:200 dilution), anti-ICOS (BV750, clone C398.4A, 1:200 dilution), anti-NK1.1 (BV711, clone PK136, 1:150 dilution), anti-CD25 (BV650, clone PC61, 1:100 dilution), anti-CD152 (BV605, clone UC10-4B9, 1:200 dilution), anti-CD62L (BV570, clone MEL-14, 1:200 dilution), anti-Granzyme B (Pacific Blue, clone GB11, 1:50 dilution), anti-Neuropilin-1 (BV421, clone 3E+12, 1:200 dilution), anti-CD103 (Biotin, clone 2E7, 1:100 dilution), anti-Helios (PE-Cy7, clone 22F6, dilution 1:30), anti-TCRβ (PE-Cy5, clone H57-597, dilution 1:300), anti-KLRG1 (BV421, clone 2F1/KLRG1, dilution 1:200), anti-KLRG1 (PE-Dazzle 594, clone 2F1/KLRG1, dilution 1:400), anti-CD38 (APC-Fire 810, clone 90, dilution 1:400), anti-CCR8 (Spark NIR 685, clone SA214G2, dilution 1:200), anti-TIM-3 (APC, clone RMT3-23, dilution 1:400), anti-TIM-3 (PE-Fire 810, clone RMT3-23, dilution 1:400), anti-CD4 (Spark NIR 685, clone GK1.5, 1:250 dilution), anti-CD206 (Alexa Fluor 700, clone C068C2, dilution 1:600), anti-F4/80 (APC/Fire750, clone BM8, dilution 1:400), anti-CD86 (PE-Dazzle 594, clone GL1, 1:1200 dilution), anti-I-A/I-E (PE-Cy5, clone M5/114.15.2, 1:2000 dilution), anti-CD90.2 (Pacific Blue, clone 30-H12, 1:500 dilution), anti-CD11b (BV510, clone M1/70, 1:1500 dilution), anti-CD64 (BV605, clone X54-5/7.1, 1:100 dilution), anti-XCR1, clone ZET, 1:300 dilution), anti-Ly6C (BV711, clone HK1.4, 1:2000 dilution), anti-CX3CR1 (BV785, clone SA011F11, 1:400 dilution), anti-T-bet (BV711, clone 4B10, 1:50 dilution), anti-IRF4 (Pacific Blue, clone IRF4.3E4, 1:100 dilution), anti-GFP (Alexa Fluor 488, clone FM264G, 1:50 dilution), anti-CD45 (PE-Fire 810, clone S18009F, 1:150 dilution), anti-Ox40 (APC-Fire750, clone Ox-86, 1:200 dilution), anti-LAG-3 (custom conjugated to NovaFluor Blue 610/70S (dye purchased from ThermoFisher), clone C9B7W, 1:300 dilution), anti-TNF (BV711, clone MP6-XT22, 1:600 dilution), anti-IL-2 (BV510, clone JES6-5H4, 1:200), anti-IL-10 (PE-Dazzle 594, clone JES5-16E3, 1:200 dilution), were obtained from BioLegend.
Anti-mouse antibodies including anti-CD69 (BUV395, clone H1.2F3, 1:100 dilution), anti-CD4 (BUV496, clone GK1.5, 1:400 dilution), anti-CD357 (BUV563, clone DTA-1, 1:400 dilution), anti-CD304 (BUV661, clone V46-1954, 1:400 dilution), anti-ST2 (BUV737, clone U29-93, 1:200 dilution), anti-CD8a (BUV805, clone 53-6.7, 1:150 dilution), anti-CD73 (BB660 custom conjugate, clone TY/23, 1:200 dilution), anti-Eomes (PE-CF594, clone X4-83, 1:100 dilution), anti-Eos (PE, clone W7-486, 1:200 dilution), anti-CD27 (R718, clone LG.3A10, 1:200 dilution), anti-Ki67 (BV480, clone B56, 1:200 dilution), anti-CD44 (BUV737, clone IM7, dilution 1:1200), anti-Ly6G (BUV563, clone 1A8, 1:700 dilution), anti-CD19 (BUV661, clone 1D3, 1:400 dilution), anti-CD45 (BUV395, clone 30-F11, 1:800 dilution, anti-CD172a (BUV395, clone P84, 1:100 dilution), anti-CD88 (BV750, clone 20/70, 1:200 dilution), anti-NK1.1 (BB700, clone PK136, 1:100 dilution), anti-Siglec-F (BB515, clone E50-2440, 1:2000 dilution) and IL-17A (PE, clone TC11-18H10, 1:600 dilution), BB630 Streptavidin (custom conjugate, 1:200 dilution) and BUV615 Streptavidin (custom conjugate, 1:200 dilution) were purchased from BD Biosciences. Anti-mouse antibodies including anti-Arginase-1 (APC, clone, dilution 1:400, A1ex5), anti-CD11c (PE-Cy5.5, clone N418, 1:1800 dilution), anti-NOS2 (PE-eFluor610, clone CXNFT, 1:800 dilution), anti-MerTK (PE-Cy7, clone DS5MMER, 1:200 dilution), anti-CD39 (PerCP-eFluor 710, clone 24DMS1, 1:400 dilution), anti-Foxp3 (PE-Cy5.5, clone FJK-16s, 1:200 dilution), anti-IFNγ (PE-Cy7, clone XMG1.2, 1:400 dilution) and anti-IL-22 (APC, clone IL22JOP, 1:200 dilution) were purchased from ThermoFisher Scientific. Anti-TCF1 (Alexa Fluor 488, clone C63D9, 1:200 dilution) was obtained from Cell Signaling technologies. Anti-TOX (PE, clone REA473, 1:200 dilution) was purchased from Miltenyi.
Anti human antibodies including anti-CD8 (BV785, clone RPA-T8, 1:100 dilution), anti-CD127 (BV605, clone A019D5, 1:50 dilution), anti-CD45 (Pacific Blue, clone HI-30, 1:200 dilution), anti-CD4 (Alexa Fluor 488, RPA-T4, 1:50 dilution), anti-CD25 (PE-Cy7, clone MA251, 1:40 dilution), anti-CD3 (APC, clone UCHT1, 1:100 dilution), anti-CD27 (BUV563, clone M-T271, 1:150 dilution) were either bought from BioLegend or BD.
anti-IL23R (unconjugated,clone EPR22838-4, 1:100 dilution) was obtained from Abcam, secondary Goat anti-rabbit antibody (Alexa Fluor 647, 1:1000 dilution) was purchased from ThermoFisher.

Validation

All antibodies used in our study are commercially available and have been titrated in-house. All antibodies have been validated by the commercial manufacturers. Validation data are available on the manufacturer`s website.

## Eukaryotic cell lines

Policy information about cell lines and Sex and Gender in Research

Cell line source(s)

B16-F10 cell line was originally received from Xenogen. MC38 cell line was received from Michael Dettmer and originally derived from American Type Culture Collection (ATCC). YUMMER1.7 cell line was purchased from Merck-Millipore.

Authentication

None of the cell lines were authenticated in these studies, for experiments cell lines with low passage numbers were used.

Mycoplasma contamination | All cell lines used in the study tested negative for mycoplasma contamination.

Commonly misidentified lines (See ICLAC register) | No commonly misidentified cell lines were used.

## Animals and other research organisms

Policy information about studies involving animals; ARRIVE guidelines recommended for reporting animal research, and Sex and Gender in Research

Laboratory animals | B6.129(Cg)-Foxp3tm3(DTR/GFP)Ayr/J (Foxp3DTR-GFP) mice and B6.129(Cg)- Foxp3tm4(YFP/icre)Ayr/J (Foxp3Cre-YFP) mice were purchased from the Jackson Laboratory (J#016959). Il23rfl/fl mice were obtained from Philip Rosenstiel (Aden et al., 2016). Il23rfl/fl mice were crossed to a Deleter Cre mouse line CMV (Deleter) Cre (J#006054) to obtain Il23rdel/del mice. IL23RtdTomato mice were generated by Mohammed Oukka and Biocytogen plasmid construction service. All mice were maintained on a C57/BL6 background and were housed in a specific-pathogen-free environment. Both female and male mice were used for experiments at the age of 6-10 weeks. Mice were socially housed with a dark/light cycle of 12h, ambient temperature of 22°C and 45-65% humidity.

Wild animals | No wild animals were used.

Reporting on sex | Findings do not only apply to one sex. Sex of the animals was considered as part of assigning experimental groups. Sex-based analyses did not reveal differences.

Field-collected samples | No field-collected samples were used in the study.

Ethics oversight | All experiments were approved by the cantonal veterinary office of Zurich.

Note that full information on the approval of the study protocol must also be provided in the manuscript.

## Flow Cytometry

### Plots

Confirm that:

☒ The axis labels state the marker and fluorochrome used (e.g. CD4-FITC).

☒ The axis scales are clearly visible. Include numbers along axes only for bottom left plot of group (a 'group' is an analysis of identical markers).

☒ All plots are contour plots with outliers or pseudocolor plots.

☒ A numerical value for number of cells or percentage (with statistics) is provided.

### Methodology

Sample preparation | Tumors were minced into small pieces and digested in RPMI 2% FCS supplemented with 1mg/ml Collagenase IV and 100 µg/ml DNAse I (both Sigma-Aldrich) at 37ºC for 45 mins. After this, the tissue was disrupted with a syringe with an 18 G needle and digested for another 15mins. After digestion, the disrupted tissue was filtered through a 100 µm cell strainer and washed with PBS. LNs and thymi were grinded through 100 µm cell strainers and washed with PBS. Immune cell enrichment was performed using mouse CD45 TIL microbeads (Miltenyi Biotec) following the manufacturer's instructions.
Ear skin was digested as previously described. In brief, skin was minced into small pieces and digested in RPMI 2% FCS supplemented with 1mg/ml Collagenase IV and 100 µg/ml DNAse I (both Sigma-Aldrich) at 37ºC for 1.5 hours. After digestion, the skin tissue was disrupted with a syringe with an 18-gauge needle and filtered through a 70-µm cell strainer.
To isolated immune cells from murine colons, 6cm long pieces from the mid-colon were collected and washed with cold PBS. Isolated tissues were incubated in HBSS (without calcium/magnesium) supplemented with 2% FCS, 10mM HEPES and 5mM DTT at 80 rpm at 37°C for 8 mins, before being incubated 3 times in HBSS (without calcium/magnesium) supplemented with 2% FCS, 10mM HEPES, 5mM EDTA at 80 rpm at 37°C for 7 mins. Next, the colons were rinsed in HBSS (with calcium/magnesium) supplemented with 2% FCS and 10mM HEPES at 80 rpm at 37 °C for 5 mins. Then, the tissues were minced using a gentleMACS™ (Miltenyi Biotec) in digestion buffer (HBSS (with calcium/magnesium) supplemented with 3% FCS, 10mM HEPES, 30ug/ml DNAse I and 100ug/ml Liberase TM) and incubated at 120 rpm at 37 °C for 25 mins before being filtered through 100-µm cell strainer and washed with cold PBS.

Instrument | Cells were analyzed using a Cytek Aurora 5L spectral flow cytometer, cells were sorted using a BD FACS Aria III 3L or 5L system.

Software | SpectroFlo (Cytek) or BD FACS Diva software was used for data acquisition, Flowjo (BD) v10 was used for data analysis.

Cell population abundance | For single cell RNA sequencing LIVE CD4+ T cells after FACS purification had a purity of >99% (based on FACS assessment).

Human LIVE CD4+CD25+CD27+CD127- Tregs had a purity of >99% after FACS purification.

Gating strategy | FSC-A and SSC-A gating was applied to exclude debris, doublets were excluded by FSC-Area vs. FSC-Height gating. Dead cells

Gating strategy

were excluded using Zombie NIR Live/Dead fixable staining reagent (BioLegend) or LIVE/DEAD Blue fixable staining reagent (ThermoFisher).
Gating strategies to identify cell populations of interest include:
γδ T cells: CD45+CD3+TCRγδ+
CD8+ T cells: CD45+CD3+CD8+CD4-
CD4+ Tcons: CD45+CD3+CD8-CD4+Foxp3-DTR-GFP-
Treg: CD45+CD3+CD8-CD4+Foxp3-DTR-GFP+ or CD45+CD3+CD8-CD4+Foxp3-Cre-YFP+
Il23r KO Treg: CD45+CD3+CD4+CD8-CD25+CD27+YFP+
Il23r WT Treg: CD45+CD3+CD4+CD8-CD25+CD27+YFP-

Human Treg: CD45+CD3+CD4+CD8-CD25+CD27+CD127-

☒ Tick this box to confirm that a figure exemplifying the gating strategy is provided in the Supplementary Information.
